# Biomimetic Planning and Design of Five-Minute Living Circle Residential Areas Inspired by Cellular Structure

**DOI:** 10.3390/biomimetics11050342

**Published:** 2026-05-14

**Authors:** Pan Pei, Yihan Wang, Feijie Xia, Yueqing Wang, Yangyang Wei

**Affiliations:** 1 Academy of Art, Yuzhang Normal University, Nanchang 330103, China; 2 School of Creative Design, Wuhan Business University, Wuhan 430056, China; 3 Architecture and Design College, Nanchang University, Nanchang 330031, China

**Keywords:** biomimetic design, biomimetic cellular structure, community planning and design, five-minute living circle, space syntax

## Abstract

Biological cellular structures exhibit a high degree of systematic organization in both morphological configuration and functional coordination, providing important biomimetic insights for urban spatial organization. To address issues in traditional high-density residential areas, such as homogeneous spatial structures and insufficient accessibility of public spaces, this study proposes a planning method for five-minute living circle residential areas based on a biomimetic cellular structure within the framework of space syntax theory. Taking a residential area in Wuhan, China, as a case study, a cell-like spatial structure model was constructed. Convex space analysis, axial analysis, and visibility analysis were conducted using Depthmap software to quantitatively evaluate key syntactic indicators, including integration, connectivity, mean depth, and choice. The results show that, compared with the original planning scheme, the biomimetic cellular planning model significantly optimized the spatial structure of the residential area by relying on the functionally synergistic mechanisms of selective permeability of the cell membrane, whole-area permeation of the cytoplasm, central regulation of the nucleus, distributed coordination of organelles, and efficient transport through cellular microfilaments. In the sample living circle, the overall integration increased from 1.27 to 1.64, the mean depth decreased from 3.79 to 3.18, and spatial connectivity increased from 3.74 to 5.44. Meanwhile, the synergy of the road network increased from 0.44 to 0.86, indicating marked improvements in spatial accessibility, connectivity, and the degree of coordination within the spatial structure. In addition, the visibility analysis showed that the pedestrian aggregation capacity of the public core space was enhanced, and the spatial vitality of public activity spaces in the residential area was improved. The findings demonstrate that the spatial organization model based on biomimetic cellular principles can effectively enhance spatial efficiency and social vitality in five-minute living circle residential areas, providing a quantifiable design method and theoretical framework for bio-inspired urban planning.

## 1. Introduction

**Figure 1:**
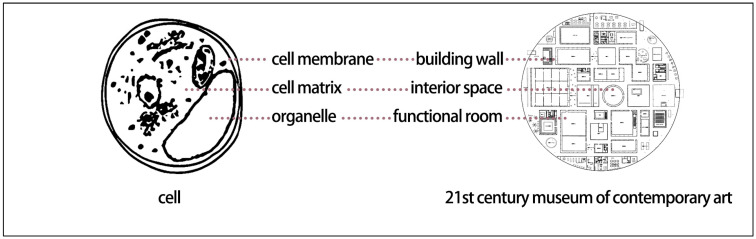
Schematic Diagram of the Analogy Between Cells and Architecture.

**Figure 2:**
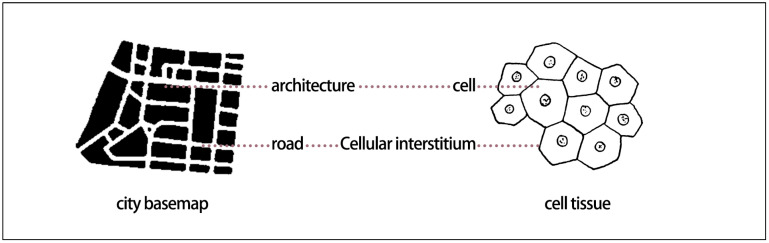
Schematic Diagram of the Analogy Between Cells and the City.

Biomimetics involves mimicking models, systems, and elements in nature to address complex human problems [1]. Cellular biomimetics has provided extensive inspiration for contemporary biomimetic design, and biomimetic cellular design research explores urban planning and architectural design methods derived from the cellular world. The cell is an efficient system that contains all the information of a living organism and is capable of metabolism, self-reproduction, and coordinated interaction with other cells. It consists of a matrix composed of water and nutrients, multiple functional components including the nucleus, internal and external transport channels, and a cell membrane [2]. The focus of this study is to explore the analogical relationships between architecture, cities, and cellular functional characteristics from a microscopic cellular perspective (Figure 1 and Figure 2). From another perspective, buildings within the urban planning system can be regarded as the “cells” that constitute the urban spatial structure, and the relationships between buildings are analogous to the interactions among cells within cellular organizations [3,4].

With the advancement of science and technology, biomimetic urban architecture has reached the forefront of urban scientific exploration. In *New Biomimetic Architecture—A New Architectural Field in the Age of Artificial Life*, Simon collected a series of design research examples that demonstrate a type of biomimetic architecture with life-like characteristics. Such architecture relies extensively on biomimetic technologies in aspects including morphological generation, component fabrication, and operational mechanisms, reflecting a new era that gives rise to “living” architecture [5]. Hensel and Menges, in *Emergent Technologies and Design: Towards a Biological Paradigm for Architecture*, explored the application of morphogenetic principles and evolutionary computation techniques to the generation and feedback of architectural and urban block forms. By integrating morphogenetic design algorithms, biomimetic engineering, and computer-aided manufacturing, they achieved comprehensive control over the design process, generating optimized buildings with integrated form, material, and structure [6]. Zaha Hadid Architects employed hair dynamics simulation in Maya software in the Kartal-Pendik Master Plan, generating path networks and urban morphology based on shortest-path algorithms. The resulting model established strong connections with surrounding urban networks and street systems [7]. Therefore, biomimetic cellular urban planning represents a meaningful and practical approach for advancing urban morphology and urban renewal.

**Figure 3:**
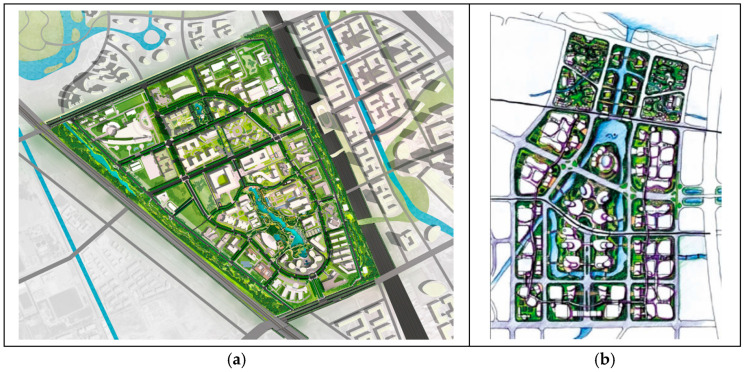
(**a**) Master Plan of Zhongguancun Life Science Park; (**b**) Conceptual Plan of Foshan International Procurement and Regional Logistics Center.

Nature-based design approaches, such as biomimetics, offer solutions for sustainable architecture and urbanism [8]. Inspired by the cellular characteristics of living systems, Forman and Godron introduced concepts such as matrix, patches, corridors, and boundaries to describe urban landscape structure and function, corresponding to the cellular matrix, organelles, circulation systems, and cell membrane [9,10]. Through cluster analysis of 15 common characteristics of hierarchical living systems, Zhang Jie et al. found that industrialized cities resemble eukaryotic cells more closely than multicellular organisms [11]. Drawing analogies between cellular characteristics and the structural and functional systems of cities, landscapes, and architecture provides a novel design approach. Landscape architect Yu Kongjian applied biomimetic design principles in the planning of the Zhongguancun Life Science Park [12], conceptualizing the site as a large-scale living organism in which buildings, landscapes, and boundaries were assigned biological roles analogous to “organelles,” “cytoplasm,” and “cell membrane,” respectively, as shown in Figure 3a. In this framework, ecological wetlands function as the cytoplasmic matrix, building clusters as organelles, transportation networks as circulation systems, and site boundaries as a cell membrane. Although the final design does not visually replicate actual cellular structures, the simulation of cellular functions results in a clear logical structure, distinct functional hierarchy, and a strong conceptual identity. This approach not only enhances the expressiveness of the planning scheme but also facilitates effective communication with users and the public. Based on cellular functional principles, Yang Changxin conceptualized the Foshan International Procurement and Regional Logistics Center as a “living cell” of an urban organism [13], aiming to establish an open and sustainably evolving ecological matrix system supported by natural conditions, along with an advanced and highly accessible public transportation network. This approach generates a clearly structured urban “living cell” capable of efficient energy, material, and information flows, as shown in Figure 3b [14]. The biomimetic cellular design strategies applied in the Zhongguancun Life Science Park and the Foshan project provide valuable references for residential area planning. By adopting such approaches, designers can create residential environments that integrate biological logic with modern functional requirements, thereby enhancing ecological performance, livability, and sustainability.

Based on the above understanding of biomimetic cellular planning patterns, and considering that the planning conditions of residential areas are consistent with the conceptual framework of science park planning, this study focuses on residential design methods. The research scope is limited to the biomimetic translation of biological cellular structures and functions, in which the constituent elements of residential areas are analogized to cellular structures. A eukaryotic cell is selected as the biomimetic prototype. A typical eukaryotic cell consists of four main components: (1) the membrane structure, a selectively permeable phospholipid bilayer that regulates the exchange of substances, such as the cell membrane and cell wall; (2) the cytoplasmic matrix, including a protein fiber network known as the cytoskeleton, which maintains cell shape, movement, and internal organization; (3) the nucleus, enclosed by a double membrane and containing most of the genetic material; and (4) organelles such as mitochondria, the endoplasmic reticulum, and the Golgi apparatus. Based on these conceptual origins and the morphological structure of eukaryotic cells, biomimetic cellular planning can be categorized into five components: (1) boundary enclosure systems analogous to the cell membrane, defining the outer limits of the planning area and regulating exchanges between internal and external environments, similar to the isolation and selective permeability of the cell membrane; (2) landscape environment systems analogous to the cytoplasm, serving as internal filling that provides support and resources for living and activities, similar to the matrix and organelles within the cytoplasm; (3) public service facilities analogous to the nucleus, functioning as the core of the planning area and responsible for management and service provision, similar to the nucleus controlling cellular activities; (4) residential clusters analogous to organelles, representing functional units such as housing, commercial, or recreational spaces, similar to mitochondria and endoplasmic reticulum; and (5) road traffic systems analogous to the cytoskeleton (microfilaments), forming the transportation network that supports the spatial structure and facilitates the movement of people and materials, similar to the cytoskeleton maintaining cellular shape and enabling movement.

Different from existing studies on biomimetic urban architecture, this study focuses on the physiological operating mechanisms of eukaryotic cell systems, including selective permeability, coordinated nucleus–cytoplasm regulation, efficient material transport, and self-organized stability. On this basis, it proposes operational design principles for the spatial organization of residential areas and establishes spatial organization rules that differ from traditional centralized and radial layouts. As the basic unit of life, the cell naturally possesses efficiency, coordination, and adaptability. Therefore, this study does not simply use the external morphology of cells as an analogy for urban architecture; rather, it translates the underlying biological mechanisms into spatial design rules for residential areas. For example, from the minimum-path and maximum-connectivity organizational logic of organelles, the filling and buffering functions of the cytoplasm, and the hierarchical control and signal-radiation mechanisms of the nucleus, quantifiable design rules are refined, including a “ring-radial hierarchical road network,” “corner-filled landscape,” and “core–cluster centripetal layout.” Ultimately, these rules jointly drive a spatial structure that differs from conventional urban architectural planning in both form and function.

In the practice of residential spatial planning, the application of space syntax has rapidly expanded, linking urban spatial analysis with transportation, land use, and human behavior studies. Increasing numbers of scholars and practitioners worldwide have applied space syntax across multiple scales, from buildings and residential areas to metropolitan regions and entire territories [15]. Space syntax is a theoretical framework used to analyze and understand the relationship between spatial organization and human behavior in architectural and urban environments. It also serves as a mathematical method for describing space, enabling quantitative analysis of spatial structures in buildings, settlements, cities, and landscapes, and examining the relationship between spatial organization and human society [16]. The theory was established by Hillier in The Social Logic of Space in the 1970s [17], followed by further developments in works such as Space is the Machine and The Common Language of Space [18,19]. Subsequent studies, including Hanson’s Decoding Homes and Houses [20], further refined and expanded the theory, opening new avenues for exploring the relationship between urban space and social structures, and gaining increasing recognition in academia.

A fundamental characteristic of space syntax theory is the integration of tangible physical spatial structures and abstract social structures into a unified analytical framework for quantitative analysis. In residential environments, this is reflected in the examination of relationships between public spaces and their social functions [21]. Ji Yang [22], based on space syntax findings and the morphological characteristics of resettlement housing, proposed regeneration strategies for existing residential areas from perspectives including residential space, transportation space, public environment, cultural and sports activities, and commercial services. Accordingly, this study adopts space syntax theory to conduct quantitative spatial analysis of residential areas. This approach employs various analytical models, with Depthmap software offering advantages in handling complex spatial networks and providing quantitative results, particularly in analyzing and visualizing spatial connectivity and integration. Roozkhosh et al. used space syntax and UCL Depthmap to calculate connectivity and integration indicators for comparing walkability across different urban areas [23]. Li applied Depthmap to analyze the spatial characteristics of suburban villages, providing references for spatial restructuring [24]. Khorsheed used depthmapX to evaluate walkability on the University of Duhok campus [25]. Numerous studies have examined accessibility in five-minute living circle residential areas using space syntax, proposing strategies such as increasing or expanding supporting facilities and enhancing road network density to improve resource sharing, demonstrating the practical significance of space syntax in residential planning. Ahmed Ali Bindajam et al. used space syntax to analyze the impact of road networks on urban structure and identify key areas for improving pedestrian accessibility [26]. These studies confirm that using Depthmap for quantitative spatial analysis of residential areas is both scientifically grounded and practically applicable.

Based on this, this study proposes a planning and design strategy for five-minute living circle residential areas based on biomimetic cellular structure and space syntax theory, aiming to optimize spatial structure and functional layout. Drawing on the structural and functional characteristics of biological cells, the study explores biomimetic cellular design methods in urban planning and architecture to create efficient, multifunctional residential environments closely aligned with residents’ daily lives. Through a case study, the proposed principles and methods are applied to actual residential planning, verifying their effectiveness and proposing optimization strategies. By integrating biomimetics and space syntax into residential planning, this study provides a new design approach that enriches the theoretical foundations of urban planning and architectural design. Through the planning and design transformation of an actual case, this study demonstrates the practical effectiveness of biomimetic cellular planning and design strategies in improving the accessibility, connectivity, and functionality of residential areas. Quantifiable and operational spatial organization rules are extracted from the underlying operating mechanisms of cellular systems. Space syntax analysis confirms that these rules can significantly increase integration, reduce mean depth, and enhance synergy, thereby verifying the effectiveness of the translation pathway from biological mechanisms to spatial rules and providing a feasible solution for urban planning practice. By using space syntax analysis tools such as Depthmap to quantitatively analyze and evaluate residential space, the scientific rigor and accuracy of the planning and design process are improved. In response to challenges posed by rapid urbanization, including spatial constraints and traffic congestion, this approach provides effective planning solutions that support healthy lifestyles, enhance residential vitality, and promote the development of more efficient, livable, and sustainable urban residential environments.

## 2. Materials and Methods

### 2.1. Overall Research Framework

**Figure 4:**
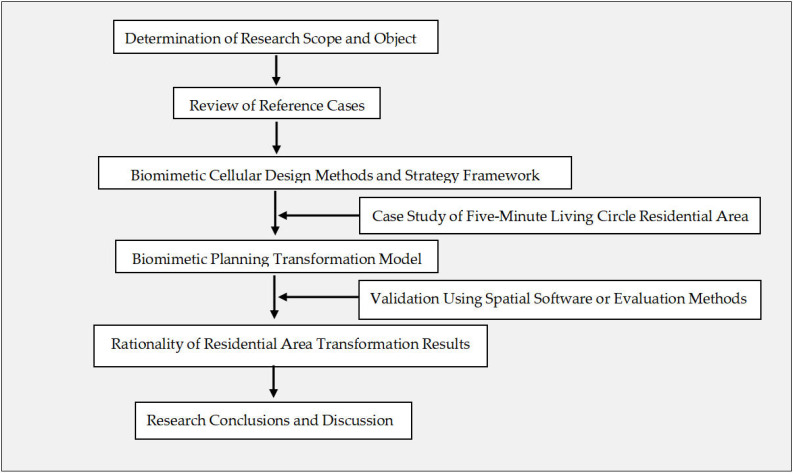
Research Workflow Diagram.

This study derives biomimetic cellular planning approaches from biomimetic urban architecture and summarizes specific planning strategies for biomimetic residential areas. Based on these strategies, a research model is established through the transformation of a residential case. The study defines its scope and object by selecting five-minute living circle residential areas as the research focus. Through the construction of a biomimetic cellular design method and strategic framework, grounded in cellular physiological structures and functional mechanisms, principles and methods for biomimetic cellular planning and design are proposed. By reviewing relevant case studies, existing biomimetic urban architecture projects are analyzed to extract biomimetic cellular design approaches. Furthermore, based on cellular structural and functional characteristics, residential planning strategies are formulated, including boundary enclosure systems, landscape environment systems, public service facilities, residential clusters, and road traffic systems. Subsequently, a biomimetic cellular residential planning model is developed through experimental design, integrating space syntax theory and employing design software such as SketchUp (version 2024) and AutoCAD (version 2025) to construct the transformation model. Finally, the rationality of the residential transformation is verified through quantitative analysis of spatial structures before and after transformation using the space syntax software Depthmap (version 0.8.0). The study adopts space syntax analysis by selecting appropriate indicators and methods for the quantitative evaluation of residential space. The experimental process includes planar model construction, grid generation, visibility analysis, and result extraction and interpretation. By comparing spatial structures before and after transformation, the effectiveness of the biomimetic cellular planning design is assessed. The overall research workflow is illustrated in Figure 4.

### 2.2. Planning Strategies for Residential Areas Based on Biomimetic Cellular Structures

**Figure 5:**
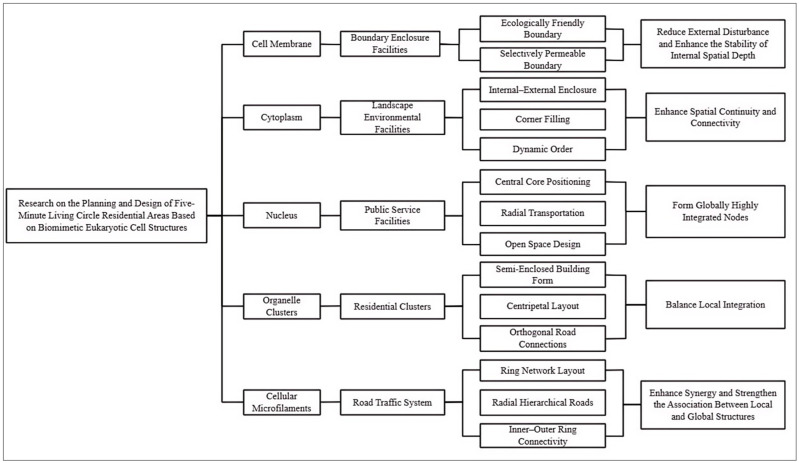
Framework of Biomimetic Cellular Planning Strategies.

**Figure 6:**
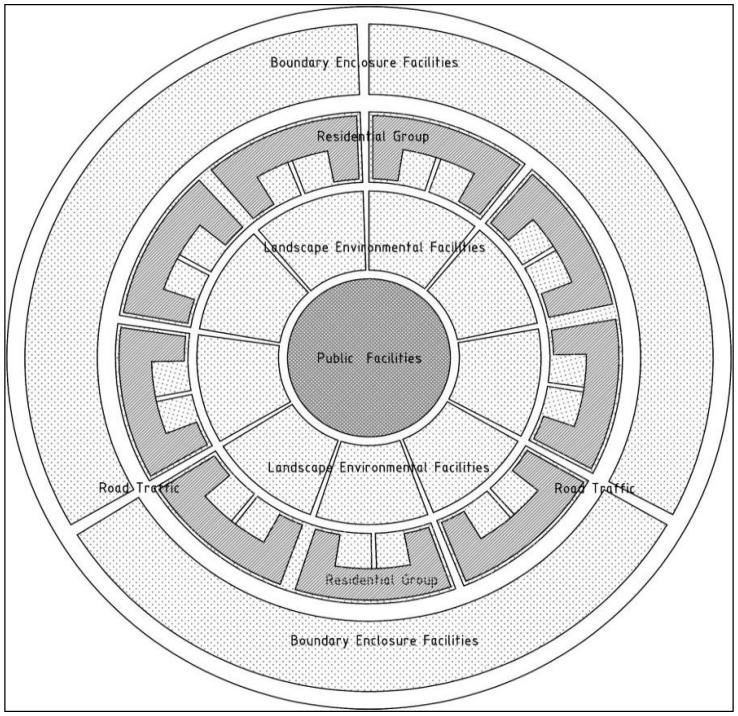
Schematic Diagram of Biomimetic Cellular Residential Planning.

Based on the basic structural components of cells, namely the cell membrane, cytoplasm, nucleus, organelle clusters, and cellular microfilaments, the fundamental constituent elements of residential areas are translated into five major parts through functional isomorphism and dynamic mechanism equivalence: boundary enclosure facilities, landscape environmental facilities, public service facilities, residential clusters, and road traffic systems (Figure 5). This translation follows the operational logic of cellular distributed autonomy coordinated with central regulation: the cell membrane enables selective permeability and dynamic regulation; the cytoplasm provides whole-area permeation and homogeneous support, thereby enhancing spatial connectivity; the nucleus undertakes core services and overall coordination, forming a globally highly integrated node; organelle clusters realize the locally efficient operation of functional units and promote the balanced distribution of local integration; and cellular microfilaments construct hierarchical transmission networks, strengthening the close association between local and global structures. Furthermore, according to the physical characteristics of cells, the spatial and functional organization of the meso-level constituent elements of residential areas is further specified, as shown in Figure 6. To clearly present the cross-scale translation logic from cellular biological mechanisms to the spatial planning of residential areas, and to quantitatively verify the scientific validity and operability of the biomimetic design, this study constructs a correspondence table linking cellular mechanisms, spatial translation rules, planning intervention measures, and expected space syntax outcomes (Table 1). This establishes a complete transmission pathway from biological prototypes to spatial forms and from qualitative conception to quantitative verification, thereby facilitating the understanding of the biomimetic planning system.

#### 2.2.1. Boundary Enclosure Facilities Analogous to the Cell Membrane

The boundary enclosure systems of residential areas are analogized to the structure of the cell membrane, serving to define internal and external divisions. They function both as interfaces that separate the residential area from the external environment and as channels for interaction and communication. This analogy ensures a balance between openness and security, privacy and connectivity, as well as esthetic and functional requirements. The cell membrane is the external boundary of the cell and performs biological functions such as serving as a physical barrier, enabling selective permeability, recognizing signals, and regulating dynamic processes. It maintains the cellular state, protects the internal cellular enclosure, selectively controls the entry and exit of substances, and acts as a medium for material and information exchange with the external environment. It is therefore a key structure through which the cell achieves a balance between openness and closure, as well as the unity of independence and coordination. Similarly, the boundary enclosure facilities of residential areas may consist of walls, fences, hedges, or other forms of physical or natural barriers, playing a role analogous to that of the cell membrane. On the one hand, they perform safety and protective functions by defining the spatial extent of the residential area, blocking external noise, environmental pollution, and unauthorized entry, maintaining the internal residential environment, and protecting residents’ safety and privacy. On the other hand, they perform functions of selective permeability and dynamic control by reasonably arranging entrances and exits, ventilation corridors, and visually permeable interfaces, thereby ensuring the orderly circulation of residents, service vehicles, materials, and information. To better align with biomimetic cellular design principles, boundary enclosure systems can integrate intelligent technologies, such as surveillance systems and automated access control, to enhance security and convenience. The design of residential boundaries can also adopt environmentally friendly materials and technologies to support sustainability goals, while reflecting cultural and esthetic values through material selection, form, and style. By dynamically regulating pedestrian flow, vehicular flow, and ecological flow within the residential area, the selective material exchange, signal recognition, and dynamic barrier functions of the cell membrane are simulated. This enables the adaptive regulation of service inputs, noise isolation, and safety services, thereby enhancing system stability and external adaptability.

(1) Ecologically friendly boundary: natural elements such as vegetation, water bodies, and rocks are used to construct boundaries, providing ecological services including biodiversity habitats, soil and water conservation, and microclimate regulation.
(2) Selectively permeable boundary: boundary enclosure design considers the regulation of pedestrian flow, vehicular movement, air circulation, and sound transmission. For example, green belts can replace solid walls to provide natural barriers while allowing airflow and visual permeability. Both visual and physical barriers can be implemented, using vegetation, landscape walls, or artistic installations as visual screens, and physical barriers such as security fencing where necessary to ensure residential safety.

#### 2.2.2. Landscape Environmental Facilities Analogous to the Cytoplasm

The landscape environmental facilities of residential areas are analogized to the cytoplasmic structure, based on the core mechanism isomorphism of systemic matrix, distributed support, whole-area permeation, and functional buffering. As a semi-fluid substance filling the space between the nucleus and organelles within the cell, the cytoplasm contains various organelles and is responsible for multiple intracellular biochemical processes. Its functions are reflected in homogeneous whole-area distribution, non-centralized dependence, the independent maintenance of local homeostasis, and the coordinated support of overall cellular operation. It can maintain microenvironmental stability and basic metabolism without relying entirely on the nucleus. Similarly, the landscape environmental facilities of residential areas, including greenery, water bodies, and resting plazas, provide residents with spaces for leisure, recreation, and social interaction. These facilities precisely correspond to the biological mechanisms of the cytoplasm, as both are organized and arranged in specific ways and support basic life activities. This analogy facilitates a deeper understanding of the complexity and dynamic nature of residential space, thereby contributing to the creation of esthetically pleasing and functionally effective living environments that meet residents’ needs. By using the continuous permeation of public spaces and landscape greening for microclimate regulation within the residential area, the dynamic processes of uniform distribution, nutrient diffusion, and regulation in the cytoplasm are simulated, thereby achieving an even distribution of landscape resources.

(1) Internal–external enclosure: similar to the cytoplasm, landscape elements form grouped enclosures at both inner and outer levels.
(2) Corner filling: under practical site conditions, areas that do not conform to the planned ring-like cellular residential structure are filled with landscape greenery.
(3) Dynamic order: landscape elements within the residential area are arranged in an orderly manner to reflect the functional efficiency of cytoplasmic space, while also accommodating temporal changes such as seasonal variation and maintaining ecosystem services and biodiversity in accordance with the dynamic ecological balance of the cytoplasm.

#### 2.2.3. Public Service Facilities Analogous to the Nucleus

The nucleus is the control center of the cell. It contains DNA and is responsible for the storage, replication, and transmission of cellular genetic information, while also serving as the regulatory center for cell division and growth. Similarly, public service facilities in residential areas, such as the comprehensive residential service center and the property management control center, function as the management and service centers of the residential area. They undertake top-level regulatory functions, including the formulation of public service rules, overall resource coordination, and service scheduling, while also carrying the culture and values of the residential area, just as the nucleus regulates the life activities of the cell. By comparing public service facilities to the nucleus, their central role within the residential area is emphasized. The design of public service facilities should reflect their identity and significance, occupy a prominent central position in the spatial layout, provide support and recovery services when the residential area faces challenges, and facilitate its development and expansion, analogous to the central position and regenerative processes of the nucleus (1.4, 2.1). By simulating the processes through which the nucleus stores information, regulates cellular metabolism, and emits directional signals, the regulatory processes of public service core radiation, coordinated resource allocation, and dynamic response to residents’ needs in the residential area are translated into spatial organization. In this way, the central area is upgraded into a whole-process regulatory hub.

(1) Central core positioning: identify the central location of the residential area as the primary concentration of public service facilities, ensuring comprehensive service coverage within a five-minute walking radius of approximately 300 m.
(2) Radial transportation: ensure accessibility to the core area by connecting all parts of the residential area through road networks and public transportation, enabling services and resources to radiate outward across the residential area, similar to the nucleus.
(3) Open space design: design open spaces such as plazas and parks within the core area to serve as venues for gathering and leisure activities for residents.

#### 2.2.4. Residential Clusters Analogous to Organelle Clusters

Organelles are specialized structures within cells that perform specific functions, such as mitochondria, the endoplasmic reticulum, and the Golgi apparatus. Each organelle carries out distinct physiological roles while collectively maintaining cellular life activities. Residential clusters are also heterogeneous “organelles” characterized by highly specialized functions and a clear division of labor. For example, various specialized service nodes in residential areas, such as elderly care service points, convenience service stations, and cultural and sports activity facilities, can rely on public service facilities within the residential area to achieve locally independent operation and distributed coordination. Some basic service nodes can even operate independently without direct interaction with the core public service facilities. This analogy emphasizes the functional performance, coordination, and sustainability of residential cluster design. The spatial distribution of residential clusters should be organized according to functional zoning, while also considering functional complementarity and accessibility within the residential area. Continuous adaptation and maintenance should be incorporated to accommodate population changes and evolving lifestyles. An efficient transportation system should be designed to reflect the spatial organization, renewal processes, and material flows analogous to organelles. The operational mode in which various specialized service nodes within residential clusters operate independently, complement one another, and provide distributed support for the overall system simulates the process by which organelles such as mitochondria and the endoplasmic reticulum perform specialized functions in a distributed manner and coordinate efficiently among units. This avoids overload caused by a single center and improves the equilibrium of the system.

(1) Semi-enclosed building form: each residential cluster adopts a C-shaped semi-enclosed configuration, creating both private and public spaces to form cohesive residential units.
(2) Centripetal layout: spatial relationships among residential clusters are organized concentrically, with a radius of approximately 300 m.
(3) Orthogonal road connections: convenient transportation links between residential clusters are ensured by using orthogonally intersecting internal roads connected directly to building entrances, facilitating daily mobility and rapid response in emergency situations.

#### 2.2.5. Road Traffic Systems Analogous to Microfilaments

Microfilaments are protein polymers that provide structural support and are involved in cellular movement, shape transformation, and material transport. Similarly, the road traffic system in residential areas provides channels for the movement of people and vehicles, ensuring both internal circulation and external connectivity, analogous to the role of microfilaments in maintaining cellular form and facilitating material transport. Microfilaments offer mechanical support, participate in signal transmission, and enable the dynamic transport of substances, forming networks that influence cellular morphology and functional spatial organization. Likewise, the road system in residential areas is responsible for the movement of residents, goods, and services, while the configuration of the road network shapes spatial layout and morphology, promoting information exchange and social interaction within and beyond the residential area. Therefore, the road traffic system can be analogized to the microfilament structure of cells. The pedestrian-priority organization, hierarchical traffic separation, shortest-path transportation, and rapid emergency evacuation in residential areas simulate the dynamic process through which cellular microfilaments provide structural support and enable directional material transport. This directly improves the transportation efficiency of the system and enables dynamic adaptation to both daily and emergency needs.

(1) Ring network layout: a circular road network is designed to traverse the entire residential area, ensuring efficient connections among residential clusters, similar to the continuous network of microfilaments.
(2) Radial hierarchical roads: different levels of roads—such as primary roads, secondary roads, branch roads, and pedestrian paths—are planned to mimic microfilaments of varying scales, accommodating diverse traffic flows and functional requirements.
(3) Inner–outer connectivity: the connectivity between the inner and outer layers of the road network is enhanced to ensure that all parts of the residential area can conveniently access the transportation system, analogous to the extensive distribution of microfilament networks.

### 2.3. Sample Residential Area Planning and Model Selection

#### 2.3.1. Five-Minute Living Circle Residential Area

According to the Code for Planning and Design of Urban Residential Areas (GB50180-2018), residential areas are hierarchically classified into fifteen-minute, ten-minute, and five-minute living circles. The concept of the “living circle” originated in Japan in the 1950s. In 1969, Japan’s National Land Agency proposed the concepts of “local living circles” and “settlement spheres” in the Third National Comprehensive Development Plan [27]. In the 1980s, South Korea introduced metropolitan living circles, regional urban circles, and rural–urban living circles in its Second National Comprehensive Development Plan [28]. Research on living circles in China began relatively early; in 1979, Taiwan introduced the concept of the “local living circle” in its urban comprehensive development plan. In 2009, Guangdong, Hong Kong, and Macao jointly compiled the first Special Plan for Building a Quality Living Circle [29]. Zhu Chasong et al. [30], taking Xiantao City as an example, proposed a hierarchical system of public service facility living circles based on residents’ travel distance, demand frequency, and service radius. Chai Yanwei et al. [31], from a spatiotemporal perspective, proposed a structure of basic living circles, commuting living circles, extended living circles, and collaborative living circles. Although living circle-based residential design has become a mainstream trend in future housing development, it is noteworthy that research on living circles remains fragmented and lacks systematic theoretical guidance [32]. Therefore, further study of current living circle design concepts has both practical significance and long-term guiding value.

As shown in Table 2, a five-minute living circle residential area accommodates a population of 5000–12,000 residents, with 1500–4000 housing units and a walking distance of approximately 300 m. It is defined as a residential area within which residents can meet their basic daily needs within a five-minute walk. Such areas are typically enclosed by branch roads or higher-level urban roads or defined by land-use boundaries, covering approximately 8–18 hectares and equipped with corresponding service facilities. Based on average walking speed, the optimal distances are approximately 750–1000 m for a 15 min living circle, 500–700 m for a 10 min living circle, and 250–300 m for a 5 min living circle [28]. Compared with larger-scale ten-minute and fifteen-minute living circles, the five-minute living circle is smaller in scale and better reflects the bottom-up characteristics of living circle planning and design, making it more relevant from a biomimetic perspective. Meanwhile, theoretical and practical studies on ten-minute and fifteen-minute living circles are relatively well developed, whereas research on five-minute living circles remains limited [33]. Therefore, this study adopts the five-minute living circle as the research scale to explore spatial openness, shared accessibility, functional organization, and social attributes under this model, providing both practical and theoretical references for future applications.

From the scale of biomolecules to that of the entire Earth, a hierarchical model of living systems has been established; however, a significant gap of several orders of magnitude remains between ecosystems (10^3^ m) and the biosphere (10^8^ m) [34]. Based on this, the super-organism hypothesis has been proposed [35,36]. By positioning the urban system (10^5^ m) within this hierarchical model, the gap between ecosystems and the biosphere is bridged, forming an arithmetic sequence of 10^3^ m, 10^5^ m, and 10^8^ m [37]. Accordingly, the concept of a “supercell city” has been proposed to replace the super-organism model [38]. The scale of a living circle residential area, approximately 10^3^ m, corresponds to a cellular block model within the cellular city framework. Considering China’s large population, the strategy of compact cellular cities [39] provides an important reference model for sustainable urban development [40].

**Table 2:** Hierarchical Scale Control of Residential Areas [41].

Distance and Scale	Fifteen-Minute Living Circle Residential Area	Ten-Minute Living Circle Residential Area	Five-Minute Living Circle Residential Area	Residential Block
Walking Distance (m)	800–1000	500	300	-
Residential Population (persons)	50,000–100,000	15,000–25,000	5000–12,000	1000–3000
Number of Housing Units	17,000–32,000	5000–8000	1500–4000	300–1000

#### 2.3.2. Transformation of a Five-Minute Living Circle Residential Area in Nanchang

The study selects a residential community at the scale of a five-minute living circle as the research object. The site is located in a district of Nanchang, in the north–central part of Jiangxi Province, China. The climate is characterized as subtropical monsoon, belonging to a hot-summer and cold-winter region, with an average annual temperature of approximately 17 °C. The residential area is bounded by a main urban road to the north, a secondary road to the east, and adjacent residential communities to the south. The primary entrance is located along the eastern road. The site benefits from convenient transportation access and daily living conditions. The total land area is 80,114 m^2^, with a total gross floor area of 163,890 m^2^, including 129,739 m^2^ above ground and 34,151 m^2^ underground. The residential area contains 14 high-rise residential buildings and one service facility building. It includes one underground parking garage, with basements used for equipment rooms and elevated ground floors serving as shared activity spaces and bicycle parking areas. The community integrates residential, leisure, and fitness functions, featuring a well-developed environment and facilities representative of modern high-quality urban housing.

**Figure 7:**
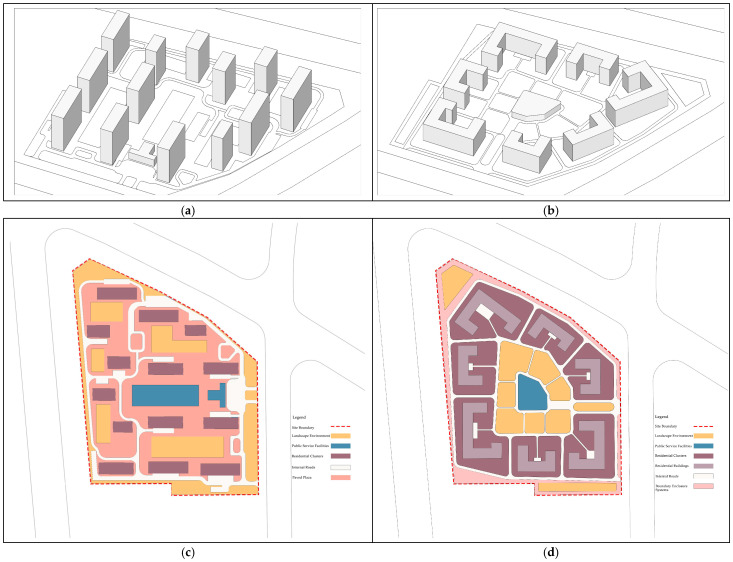
(**a**) Axonometric View of the Residential Area Before Transformation; (**b**) Axonometric View of the Residential Area After Transformation; (**c**) Master Plan of the Residential Area Before Transformation; (**d**) Master Plan of the Residential Area After Transformation.

Field investigation and analysis indicate that the community generally conforms to the scale of a five-minute living circle, with a typical high-rise linear layout, as shown in Figure 7a. However, several common issues associated with high-rise residential developments were identified: high-voltage power lines within the site negatively affect the landscape; limited internal traffic space restricts efficient mobility; the building layout does not fully utilize landscape resources and exhibits single functional typologies; supporting facilities are insufficient, overly standardized, lacking diversity and identity, and unable to meet certain service needs; the lower-quality residential area to the south and high population density may raise concerns regarding living space and privacy; simplistic boundary design results in noise disturbance from surrounding arterial roads; and the planning of the living circle is overly function-oriented, potentially neglecting community cohesion and neighborhood interaction, as illustrated in Figure 7c.

Based on the biomimetic cellular planning framework, a redesign is proposed. While maintaining the overall site area, form, primary entrance location, and total building area, building heights are reduced and building density is increased, as shown in Figure 7b. A C-shaped building prototype is adopted and arranged in a centripetal configuration, forming a semi-enclosed spatial structure centered on public service facilities, with a service radius of 250–300 m corresponding to a five-minute walking distance, as illustrated in Figure 7d. The overall layout forms a multi-layered structure of inner and outer rings, corresponding to the hierarchical organization of the nucleus–cytoplasm–cell membrane. Compared with conventional high-rise linear residential layouts, the biomimetic cellular configuration exhibits stronger inward orientation, enhanced safety and privacy, improved internal landscape orientation, and a more harmonious urban interface due to the reduced building height.

### 2.4. Space Syntax Analysis

#### 2.4.1. Space Syntax Methods

Based on different requirements, space syntax abstracts space into three types: point, line, and plane. Points correspond to origins, pixels, and grid cells; lines correspond to axial lines, segments, natural streets, and road centerlines; and planes correspond to blocks, convex spaces, and isovist fields. Within the framework of space syntax theory, space possesses both spatial and social attributes. Straight-line movement of individuals exhibits linear characteristics; interpersonal interaction requires mutual visibility and involves staying behavior, thus exhibiting planar characteristics; and when observing space, individuals take their standing position as a point and radiate their line of sight outward, thus exhibiting point-based characteristics. These three spatial characteristics are fundamentally distinct [42]. Therefore, based on human behavioral characteristics and spatial attributes, space can be divided into three types: convex space, axial lines, and isovists. Convex space analysis, axial analysis, and visibility analysis are the three primary analytical methods in space syntax theory, used to quantify and evaluate the structural characteristics of urban space [43]. Convex space refers to the set of all spatial areas that can be directly seen from a given point. This method focuses on visibility and connectivity, evaluating spatial openness and accessibility by calculating the size and shape of convex spaces. It can be used to identify central and peripheral areas and to assess spatial integration. Axial analysis interprets spatial structure and movement by identifying and analyzing axial lines, which represent primary movement paths such as streets, corridors, or other linear spatial elements. By measuring the length, direction, and connectivity of axial lines, spatial depth, orientation, and overall layout can be evaluated. Visibility analysis focuses on the areas visible from one or multiple observation points. This method is commonly used to assess visibility ranges and the spatial characteristics observable from specific viewpoints, and can be applied to evaluate accessibility, surveillance potential, and visual connectivity.

**Figure 8:**
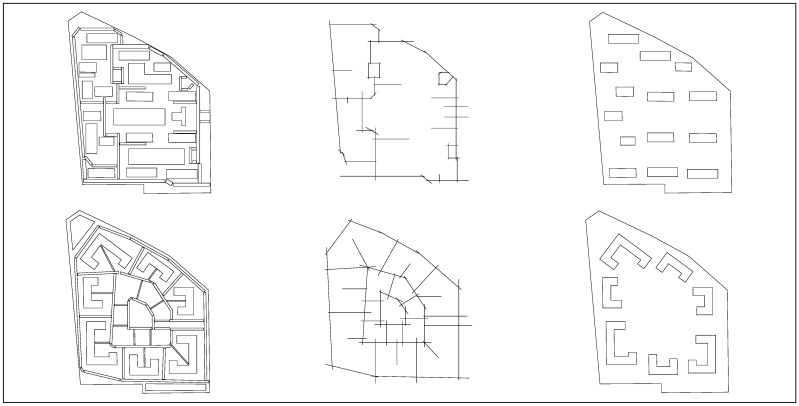
Pre-processing Diagram for Space Syntax Analysis.

This study employs the space syntax software Depthmap+ Beta 1.0 to conduct visualized analysis of spatial relationships. Depthmap is one of the most mature tools for spatial structure analysis, capable of generating a series of parameters used as evaluation indicators for residential accessibility and related aspects, thereby quantitatively reflecting the accessibility, openness, and interaction level of biomimetic cellular planning spaces. Key indicators selected include integration, mean depth, and connectivity. The experimental process using Depthmap for convex space analysis, axial analysis, and visibility analysis consists of five stages: first, collection of overall site plan data, including building layouts and street network satellite maps; second, construction of a planar DXF model by drawing spatial layouts, road networks, and building configurations in CAD (Figure 8), exporting them as DXF files and importing them into Depthmap; third, grid generation, in which an appropriate grid density (set to 3000) is selected according to the spatial scale and the study area is filled; fourth, data analysis, where parameters are configured—such as selecting a topological radius of R = 3 based on the activity range of a five-minute walking distance—for local integration analysis, enabling the calculation of syntactic attributes of the spatial network, including connectivity, integration, and depth values; and fifth, result extraction, where after conducting convex space, axial, and visibility analyses, the software outputs the results in CSV table and PNG formats.

#### 2.4.2. Space Syntax Variables

In space syntax analysis, connectivity, control value, depth, choice, integration, clustering, synergy, intelligibility, and pedestrian interface value are key quantitative indicators used to evaluate and understand the topological characteristics of urban space and patterns of human behavior. When a spatial system exhibits strong clustering, it is characterized by high integration, low depth, and strong accessibility, indicating that spatial units are closely connected with minimal barriers between them; conversely, lower integration and higher depth suggest that spatial units are more dispersed and separated by greater obstacles [44].

(1) Connectivity: Connectivity refers to the degree of linkage between nodes in a spatial network, representing the number of spaces directly connected to a given node. A higher connectivity value indicates that a space is linked to more adjacent spaces, reflecting stronger spatial interaction, easier access to surrounding spaces, and greater permeability [45,46].

The calculation formula is shown in Equation (1):

(1) $$C_{i}=k$$

where $C_{i}$ represents the connectivity value of node i, and k denotes the number of nodes directly connected to node i.

(2) Control Value: Control value reflects the degree to which a spatial node controls movement within the network and is generally related to its connectivity. A higher control value indicates that a space has a broader field of view, allowing greater visibility and supervision over the spatial system, and thus occupies a relatively more important position within its surrounding area. The calculation formula is shown in Equation (2):

(2) $$Ctr\;l_{i}\Sigma_{j=1}^{k}=\frac{1}{C_{j}}$$

where $Ctr\;l_{i}$ represents the control value of node *i*; *j* (*j* = 1,2, …, *k*) denotes the nodes directly connected to node i; $C_{i}$ represents the connectivity value of node *j*; and *k* is the number of nodes directly connected to node *i*.

(3) Choice: Choice measures the number of alternative paths available from a given node, reflecting the diversity of routes and the potential for movement. A higher choice value indicates that a space is more likely to be traversed by pedestrian flow and has greater potential attractiveness for movement.
(4) Depth: Depth represents the “distance” from the outermost part of the network to a specific node, defined as the shortest path length from one node to the farthest node in the network. Mean depth refers to the average depth of a space within the spatial system. A higher mean depth value indicates that the space is farther from other spaces and requires more steps to reach them, implying lower accessibility and convenience. The calculation is shown in Equation (3):

(3) $$Di=\Sigma_{i=1}^{n}d_{ij}$$

where Di represents the depth value of node i; j (j = 1, 2, …, n) denotes the nodes connected to node i; $d_{ij}$ represents the shortest distance from node iii to node j; and n is the total number of nodes in the connectivity graph.

The calculation formula for mean depth is shown in Equation (4):

(4) $$MD_{i}=\frac{\Sigma_{j=1}^{n}d_{ij}}{n-1}$$

where $MD_{i}$ represents the mean depth value of node i; j (j = 1, 2, …, n) denotes the nodes connected to node iii; $d_{ij}$ represents the shortest distance from node i to node j; and n is the total number of nodes in the connectivity graph.

(5) Integration: Integration evaluates the centrality of nodes within a spatial network. Nodes with higher integration values are more centrally located within the network. The calculation formulas are shown in Equations (5) to (8):

(5) $$RA_{i}=\frac{2\left(MD_{i}-1\right)}{n-2}$$

(6) $$RRA_{i}=\frac{RA_{i}}{D_{n}}$$

(7) $$Dn=\frac{2\left(n\left(log_{2}\frac{n+2}{3}-1\right)+1\right)}{\left(n-1\right)\left(n-2\right)}$$

(8) $$I_{i}=\frac{1}{RRA_{i}}$$

where $RA_{i}$ represents the relative asymmetry of node i, obtained through the first normalization using $MD_{i}$; $RRA_{i}$ represents the real relative asymmetry of node i, obtained through the second normalization using Dn; Dn is the normalization parameter; $I_{i}$ represents the integration value of node i; $MD_{i}$ is the mean depth value of node i; and n is the total number of nodes in the connectivity graph.

A higher integration value of a given space indicates a shorter distance to other spaces, less influence from obstacles, and thus better accessibility and convenience, which correspond to higher pedestrian density and activity levels. Integration is generally divided into global integration and local integration: global integration considers the relationships between a given space and all other spaces within the entire system, while local integration considers only the relationships within a limited topological distance (typically three steps) between a given space and other spaces [46,47].

(6) Aggregation: Aggregation represents the degree to which certain spatial features or activities are concentrated and is used to evaluate the clustering characteristics of space. A higher visual aggregation value of a given space indicates stronger constraints imposed by surrounding spatial interfaces, resulting in a more restricted field of view and a higher sense of spatial enclosure. The calculation formula is shown in Equation (9):

(9) $$Visual\;Clustering\;Coefficient_{\left(i\right)}=\frac{k}{k\left(k-1\right)}$$

where $Visual\;Clustering\;Coefficient_{\left(i\right)}$ represents the visual aggregation value of node *i*, and k denotes the number of nodes contained within the area where node i is located.

According to space syntax theory and related studies, the value range of visual aggregation is 0–1.0 [48]. The closer the value is to 1.0, the greater the degree of visual obstruction and constraint within the space; when the value equals 1.0, it indicates that the space is a convex space [49].

(7) Synergy: Synergy measures the interaction and coordination among different spatial elements, reflecting the degree of spatial integration. A higher synergy value of a given space indicates a stronger relationship between local spatial structure and the overall spatial system. The calculation formula is shown in Equation (10):

(10) $$R^{2}=\frac{{\left(\Sigma_{i=1}^{n}\left(I_{3}-{\bar{I}}_{3}\right)\left(I_{i}-\bar{I}\right)\right)}^{2}}{\Sigma_{i=1}^{n}{\left(I_{3}-{\bar{I}}_{3}\right)}^{2}{\left(I_{i}-\bar{I}\right)}^{2}}$$

where R represents the synergy value of node i; $I_{3}$ represents the three-step integration of node *i*; ${\bar{I}}_{3}$ represents the average three-step integration of all nodes; $I_{i}$ represents the global integration of node *I*; $\bar{I}$ represents the average global integration of all nodes; and n is the total number of nodes in the connectivity graph.

According to space syntax theory and related studies, when the coefficient of determination $R^{2}$ ranges from 0 to 0.5, the spatial system exhibits low synergy, indicating weak correlation between local and global spatial structures, and the overall space tends to present an axial structure with multiple integration cores. When $R^{2}$ ranges from 0.5 to 0.7, the spatial system shows relatively high synergy, with good coordination between local and global spatial structures. When $R^{2}$ ranges from 0.7 to 1.0, the spatial system demonstrates very high synergy, indicating a strong relationship between local and global spatial structures, with highly coordinated organization, and the overall space tends to exhibit an axial structure with a single integration core.

(8) Comprehensibility: Comprehensibility evaluates the clarity and intelligibility of spatial layout, influencing human spatial cognition. A higher comprehensibility value indicates that spaces with higher local connectivity also exhibit higher global integration, suggesting that the spatial system is clear, intelligible, and well-structured. The calculation formula is shown in Equation (11):

(11) $$R^{2}=\frac{{\left(\Sigma_{i=1}^{n}\left(C_{i}-\bar{C}\right)\left(I_{i}-\bar{I}\right)\right)}^{2}}{\Sigma_{i=1}^{n}{\left(C_{i}-\bar{C}\right)}^{2}{\left(I_{i}-\bar{I}\right)}^{2}}$$

where R represents the comprehensibility of node iii; $C_{i}$ represents the connectivity value of node i; $\bar{C}$ represents the average connectivity value of all nodes; $I_{i}$ represents the global integration of node i; $\bar{I}$ represents the average global integration of all nodes; and n is the total number of nodes in the connectivity graph.

According to space syntax theory and related studies, when the coefficient of determination $R^{2}$ ranges from 0 to 0.5, the spatial system exhibits low comprehensibility, indicating that the spatial structure is difficult for pedestrians to understand, and it is challenging to perceive the overall structure from local spatial information, resulting in low spatial legibility; when $R^{2}$ ranges from 0.5 to 0.7, the spatial system shows relatively high comprehensibility, meaning that the spatial structure is easier for pedestrians to understand, with higher spatial legibility; when $R^{2}$ ranges from 0.7 to 1.0, the spatial system demonstrates very high comprehensibility, indicating that the spatial structure is very easy for pedestrians to understand, with very high spatial legibility.

(9) Pedestrian Interface Value: The pedestrian interface value measures the level of activity along pedestrian interfaces and is generally related to commercial activities and pedestrian density. A higher pedestrian interface value indicates a stronger capacity of a space to accommodate both internal movement and external through-flow of pedestrians, and a lower likelihood of traffic congestion. The calculation formula is shown in Equation (12):

(12) $$R^{2}=\frac{{\left(\Sigma_{i=1}^{n}\left(C_{i}-\bar{C}\right)\left(I_{i}-\bar{I}\right)\right)}^{2}}{\Sigma_{i=1}^{n}{\left(C_{i}-\bar{C}\right)}^{2}{\left(I_{i}-\bar{I}\right)}^{2}}$$

where R represents the pedestrian interface value of node iii; $C_{i}$ represents the choice value of node i; $\bar{C}$ represents the average choice value of all nodes; $I_{i}$ represents the global integration of node i; $\bar{I}$ represents the average global integration of all nodes; and n is the total number of nodes in the connectivity graph. According to space syntax theory and related studies, when the coefficient of determination $R^{2}$ ranges from 0 to 0.5, the spatial system exhibits a low pedestrian interface value, indicating weak capacity to attract and accommodate pedestrian flow and a low likelihood of traffic congestion; when $R^{2}$ ranges from 0.5 to 0.7, the spatial system shows a relatively high pedestrian interface value, indicating stronger capacity to attract pedestrian flow and a higher likelihood of congestion; when $R^{2}$ ranges from 0.7 to 1.0, the spatial system demonstrates a very high pedestrian interface value, indicating strong capacity to concentrate pedestrian flow and a high likelihood of traffic congestion [50].

#### 2.4.3. Social and Spatial Attributes of Space Syntax Parameters in Residential Areas

**Figure 9:**
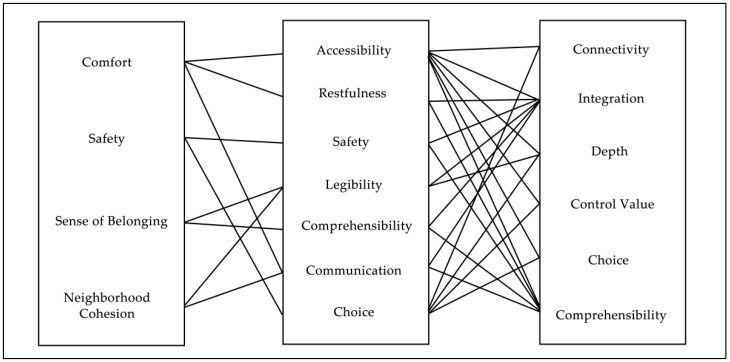
Relationship Between Space Syntax Parameters and Social Attributes [52].

The primary parameters in space syntax include integration, connectivity, choice, depth, and comprehensibility. These parameters not only represent the spatial attributes of nodes but also implicitly reflect the social attributes of space, such as accessibility, usability, communicability, safety, and wayfinding [51]. When engaging in outdoor activities, people exhibit physiological, psychological, and behavioral characteristics in their spatial choices, which in turn indirectly reflect the social attributes of space. These social attributes reveal the interactive relationship between people and space. Based on space syntax variables, a syntactic evaluation system for public activity spaces in five-minute living circle residential areas can be established, as shown in Figure 9.

To quantitatively reflect the social attributes of space as well as the overall spatial structure of residential areas—including the permeability, accessibility, convenience, and degree of enclosure of internal roads and residential buildings—this study refers to the selection of syntactic indicators for residential areas proposed by Liu Tong [52] and Ji Yang [22]. In convex space analysis, integration, mean depth, and connectivity are selected as syntactic indicators; in axial analysis, integration, choice, and control value are selected; and in visibility analysis, visual integration, connectivity, control value, clustering coefficient, and agent-based simulation (agent) are selected as syntactic indicators.

## 3. Results

### 3.1. Convex Space Analysis

**Figure 10:**
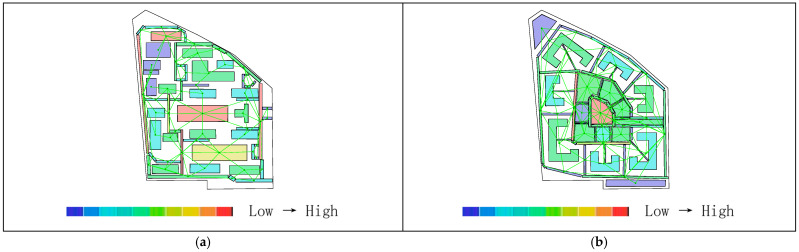
(**a**) Convex Space Base Map of the Residential Area Before Transformation; (**b**) Convex Space Base Map of the Residential Area After Transformation.

As shown in Table 3, the global integration values before transformation range from 0.88 to 1.52, with an average value of 1.27. The local integration values before transformation range from 1.00 to 2.47, with a relatively small distribution span and an average value of1.74. Based on the activity range of residents, a topological radius of R = 3 is selected to analyze local integration within the living circle. The results show that the local integration values before transformation range from 1.00 to 2.47, with an average value of 1.74. The integration analysis of the convex space base map is shown in Figure 10.

**Figure 11:**
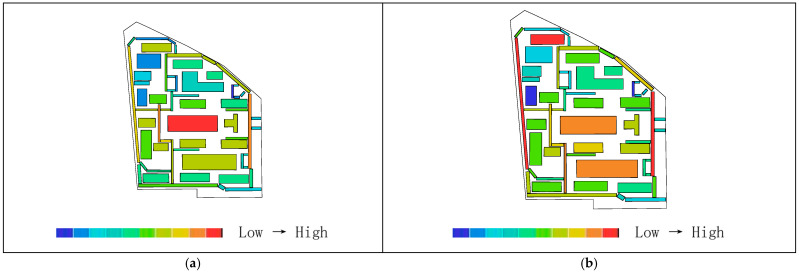
(**a**) Global Integration of Convex Space Before Transformation; (**b**) Local Integration of Convex Space Before Transformation.

**Figure 12:**
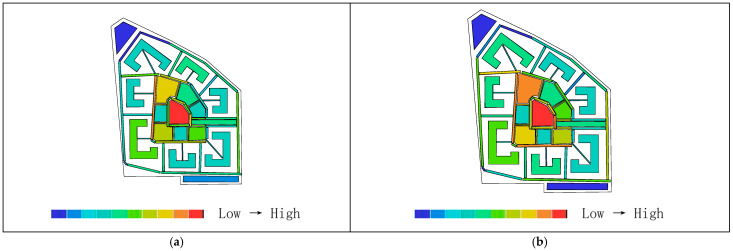
(**a**) Global Integration of Convex Space After Transformation; (**b**) Local Integration of Convex Space After Transformation.

As shown in Figure 11a, the highest global integration is located in the central plaza space, while Figure 11b indicates that the highest local integration is found at the northwestern corner of the residential buildings and along the internal roads on the eastern and western sides, followed by the central plaza and the southern landscape area. As shown in Table 2, after transformation, the global integration ranges from 1.16 to 2.25, with an average value of 1.64, and the local integration ranges from 1.54 to 2.75, with an average value of 2.12, both showing relatively higher values overall. As shown in Figure 12a,b, the areas with the highest integration are located in the core public service facilities. The nucleus-like layout produces a distribution pattern in which overall integration gradually decreases from the center toward the periphery while radiating evenly, rather than exhibiting the excessively high central values and low peripheral values often found in conventional centralized layouts. Therefore, the transformed scheme shows higher overall spatial integration than the pre-transformation scheme, indicating better accessibility and convenience. Correspondingly, the core public area also demonstrates higher pedestrian density and activity levels. Moreover, through the combined effect of radial roads and a ring-shaped structural framework, spatial integration can permeate evenly throughout the entire residential area, demonstrating both topological rationality and support from biological cellular mechanisms.

**Figure 13:**
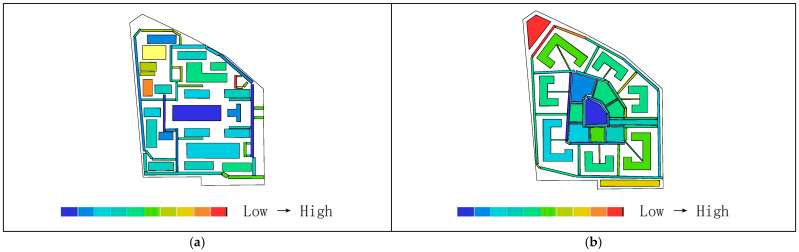
(**a**) Mean Depth of Convex Space Before Transformation; (**b**) Mean Depth of Convex Space After Transformation.

As shown in Table 2, the average depth before transformation ranges from 3.23 to 4.17, with an average value of 3.79. As shown in Figure 13a, the areas with higher average depth are mainly concentrated on the small path on the northeastern boundary of the living circle. According to Table 2, the average depth after transformation ranges from 2.57 to 4.03, with an average value of 3.18. Figure 13b shows that the warmest areas are located in the landscape and roads at the northwest corner, while the coldest areas are in the central region. Therefore, the average depth before transformation is higher than that after transformation, indicating that in the overall space of the residential area, the distance between spaces was greater before the transformation, requiring more steps to reach other spaces, which resulted in weaker accessibility and convenience.

**Figure 14:**
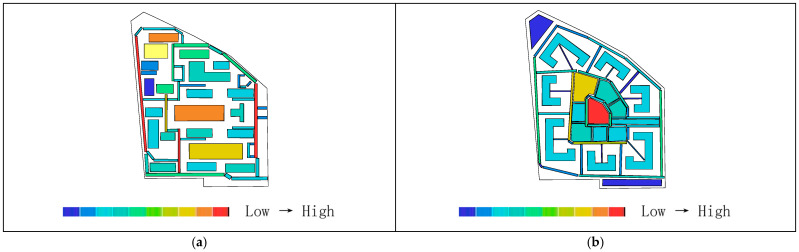
(**a**) Connectivity of Convex Space Before Transformation; (**b**) Connectivity of Convex Space After Transformation.

As shown in Figure 10a, the original residential area can be divided into 61 convex spaces. Figure 14a shows that the areas with the highest connectivity (indicated by the warmest colors) are the internal roads on the east and west sides, with a value of 9, meaning that they are connected to 9 surrounding buildings and roads. This is followed by the central public plaza and the residential buildings on the northwest side, both with a value of 5. As shown in Figure 14b, the transformed residential area contains 64 convex spaces, with an average connectivity value of 5.43, which is higher than the pre-transformation average of 3.74. The highest connectivity value after transformation is located at the central public service facilities, with a value of 14, while the lowest connectivity values are found in the green landscape areas at the northwest and southeast corners due to their distance from the central area. The standard deviation is 2.20, slightly higher than the pre-transformation value of 1.87, indicating a clearer hierarchical distribution of connectivity. The semi-enclosed building form of residential clusters, analogous to organelle clusters, increases the number of direct connections among clusters, resulting in an overall improvement in connectivity rather than the strengthening of only local nodes. Through the synergistic mechanisms of distributed operation of organelle-like units, whole-area filling by the cytoplasmic matrix, and hierarchical transmission through the cellular microfilament network, an organizational model is constructed that combines direct interconnection among clusters with centrally coordinated linkage. Therefore, the post-transformation connectivity is generally higher than that before transformation; that is, the location of public service facilities is connected to more roads and residential clusters, indicating a higher degree of linkage, better spatial permeability, and a clearer hierarchical distribution.

In summary, the convex space analysis indicates that the pre-transformation residential spatial structure consists of multiple high-rise buildings arranged in an orderly manner primarily for economic efficiency, with relatively weak interconnections between buildings. Residents need to undergo more spatial transitions to move from one point to other spaces, resulting in poor connectivity and accessibility, as well as insufficient openness of internal spaces. In addition, outdoor public facilities exhibit single functions and constrained spatial distribution, leading to low legibility and intelligibility, which in turn weaken neighborhood interaction and reduce community cohesion. The central axis from the sole entrance to the central plaza, together with the peripheral roads, serves as the main connection route, and ites limited accessibility may lead to difficulties in fire evacuation for high-rise buildings. In contrast, after transformation, the centripetal residential spatial structure demonstrates improved accessibility and convenience in both the overall space and core public areas, enhanced spatial permeability, higher pedestrian density and activity levels, and a more clearly defined hierarchical spatial organization.

### 3.2. Axial Analysis

Areas with higher axial integration values are represented by warmer colors, while areas with lower values are shown in cooler colors. As shown in Table 4, before transformation, the global integration ranges from 0.48 to 0.84, with an average value of 0.65.

**Figure 15:**
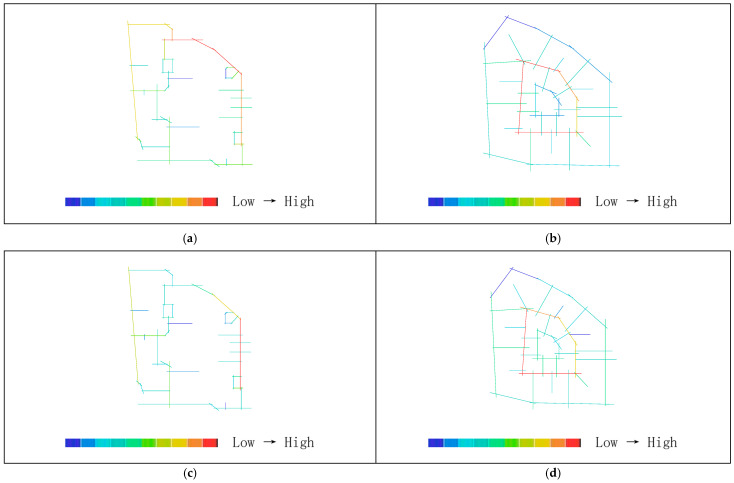
(**a**) Global Axial Integration Before Transformation; (**b**) Global Axial Integration After Transformation; (**c**) Local Axial Integration Before Transformation; (**d**) Local Axial Integration After Transformation.

Figure 15a indicates that the highest integration of the original road network is located at the northeast corner near the main entrance of the residential area, while the lowest values occur on two proximal roads near the central plaza. As shown in Table 3, the local integration before transformation ranges from 0.58 to 2.25, with an average value of 1.13. Figure 15c shows that the highest value is on the road at the eastern main entrance, while the lowest values are on the two proximal roads near the central plaza. After transformation, as shown in Table 3, the global integration ranges from 0.88 to 2.10, with an average value of 1.33, representing an increase of 0.68 compared to the pre-transformation condition. Figure 15b shows that the highest integration values are located in the central ring area near residential clusters and public service facilities, while the lowest values occur on the outer ring roads on the north and east sides. Table 3 indicates that the local integration after transformation ranges from 1.04 to 2.40, with an average value of 1.57, representing an increase of 0.44. Figure 15d shows that the highest local integration is also located in the central ring area near residential clusters and public service facilities, while the lowest values are found on the northern outer ring roads. Therefore, the higher local integration compared to global integration before transformation indicates strong connectivity among roads within local areas, while after transformation, the overall axial integration is higher, suggesting improved efficiency of both pedestrian and vehicular movement.

**Figure 16:**
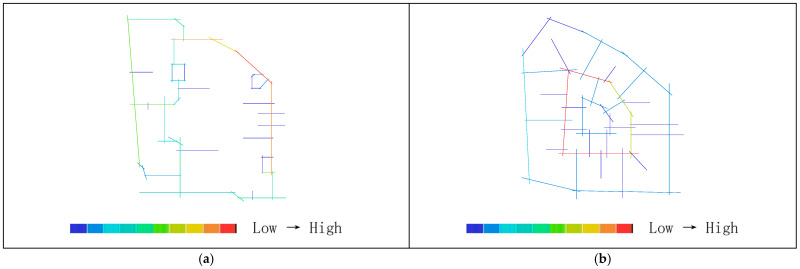
(**a**) Axial Choice Before Transformation; (**b**) Axial Choice After Transformation.

Areas with higher axial choice values are represented by warmer colors, while areas with lower values are shown in cooler colors. As shown in Table 3, before transformation, the road choice values range from 0 to 750, with an average value of 203.41. Figure 16a indicates that the roads with the highest choice values are located near the main entrance on the eastern and northeastern sides. After transformation, the road choice values range from 0 to 480, with an average value of 88.10. Figure 16b shows that the highest choice values are found on the three roads located on the north, west, and south sides of the central ring area, near residential clusters and public service facilities. Comparing the visualized data, it can be observed that before transformation, the roads near the main entrance have the highest choice values and thus the greatest potential to attract through traffic, whereas after transformation, the overall choice values of the residential roads are generally lower, with the highest pedestrian movement concentrated on the roads surrounding the central public plaza.

**Figure 17:**
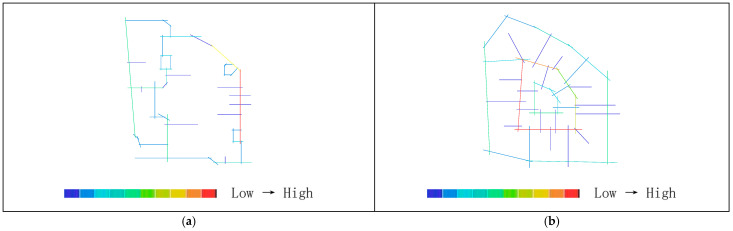
(**a**) Axial Control Value Before Transformation; (**b**) Axial Control Value After Transformation.

Areas with higher axial control values are represented by warmer colors, while areas with lower values are shown in cooler colors. As shown in Table 3, before transformation, the control values of the residential road network range from 0.14 to 5.09, with an average value of 1.00. Figure 17a indicates that the highest control value is located on the eastern road near the main entrance. After transformation, the control values range from 0.13 to 3.77, with an average value of 1.00. Figure 17b shows that the highest control values are found on the three roads located on the north, west, and south sides of the central ring area, near residential clusters and public service facilities. Comparing the distribution of warm colors in the visualizations indicates that before transformation, the road at the main entrance has the highest control value, offering better visibility and a stronger ability to oversee and control the entire residential space, whereas after transformation, the roads in the central ring area exhibit the best visual control.

**Figure 18:**
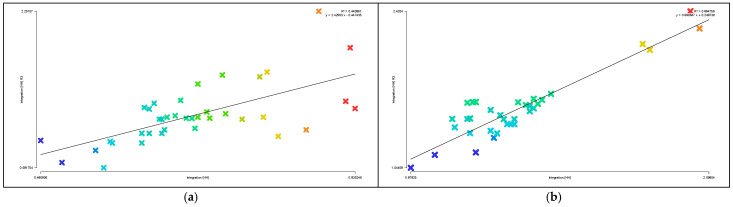
(**a**) Axial Synergy Before Transformation (x = Integration [HH], y = Integration [HH] R3, R^2^ = 0.44); (**b**) Axial Synergy After Transformation (x = Integration [HH], y = Integration [HH] R3, R^2^ = 0.86).

As shown in Figure 18a,b, the synergy before transformation is R^2^ = 0.44, while after transformation it is R^2^ = 0.86, representing an increase of 0.42. This indicates that the post-transformation residential area exhibits higher synergy, meaning a stronger correlation between local and global spatial structures. Pedestrian flow on the central ring roads can more easily converge with the overall movement, making it easier to identify core spaces and distribute flows through radial branch roads, thereby achieving a better balance between openness and enclosure.

**Figure 19:**
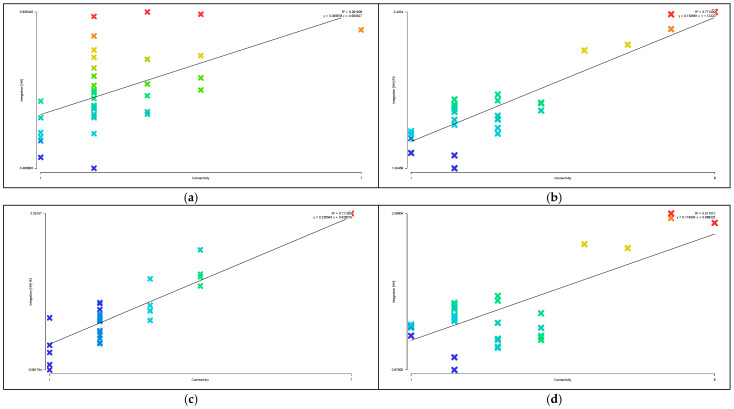
(**a**) Axial Comprehensibility Before Transformation (x = Connectivity, y = Integration [HH], R^2^ = 0.28); (**b**) Axial Comprehensibility After Transformation (x = Connectivity, y = Integration [HH], R^2^ = 0.77); (**c**) Axial Comprehensibility Scatter Plot Before Transformation (x = Connectivity, y = Integration [HH] R3, R^2^ = 0.71); (**d**) Axial Comprehensibility Scatter Plot After Transformation (x = Connectivity, y = Integration [HH] R3, R^2^ = 0.52).

As shown in Figure 19a,c, before transformation, the global comprehensibility is R^2^ = 0.28, which is less than 0.5, while the local comprehensibility is R^2^ = 0.71, which is greater than 0.7. As shown in Figure 19b,d, after transformation, the global comprehensibility is R^2^ = 0.77, which is greater than 0.7, and the local comprehensibility is R^2^ = 0.52, which is greater than 0.5. This indicates that before transformation, the overall comprehensibility is low, making the spatial structure difficult to understand and resulting in low spatial legibility, while local legibility is relatively high. After transformation, both global and local spatial legibility are improved, indicating higher internal and external spatial permeability within the residential area.

**Figure 20:**
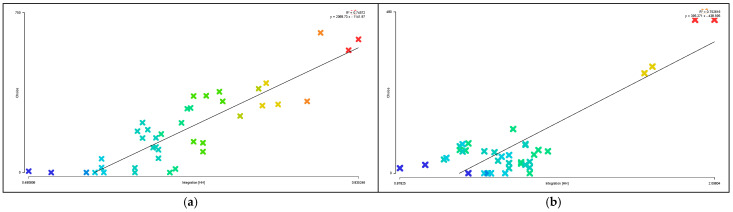
(**a**) Axial Pedestrian Interface Value Before Transformation (x = Choice, y = Integration [HH], R^2^ = 0.74); (**b**) Axial Pedestrian Interface Value After Transformation (x = Choice, y = Integration [HH], R^2^ = 0.75).

As shown in Figure 20a,b, the pedestrian interface value before transformation is R^2^ = 0.74, while after transformation it is R^2^ = 0.75. The two values are nearly equal, both exceeding 0.7, indicating that the spatial system has a strong capacity to accommodate both internal and external pedestrian flows, with generally smooth traffic conditions. This suggests a high degree of alignment and coexistence between through-movement and internal residential movement within the area.

The axial analysis indicates that, before transformation, the road system of the residential area contains a large number of vehicular dead-end roads and turning spaces, with vehicle flow primarily directed toward underground parking facilities located around the perimeter. The main vehicular traffic follows the outer ring roads, while pedestrian movement is concentrated along the central axis from the main entrance to the public plaza. This results in high traffic density at the primary entrance, where pedestrian and vehicular flows intersect. The two main entrances are both located on the eastern side, forcing residents in more distant buildings to rely on peripheral vehicular roads or indirect routes through paved plazas to access public spaces and entrances, leading to overly concentrated control and limited route choice. In contrast, compared with the simple ring road system before transformation, the redesigned road network prioritizes pedestrian movement as the dominant mode of transportation, with activity confined within the five-minute living circle radius. The hierarchical road system, consisting of inner and outer rings combined with radial distribution, forms a three-level network that improves the efficiency of both pedestrian and vehicular circulation. The core public area achieves higher legibility and a broader visual field, while the highest pedestrian choice values are concentrated on roads surrounding the central public space, enhancing the utilization and distribution efficiency of public facilities.

### 3.3. Visibility Analysis

**Figure 21:**
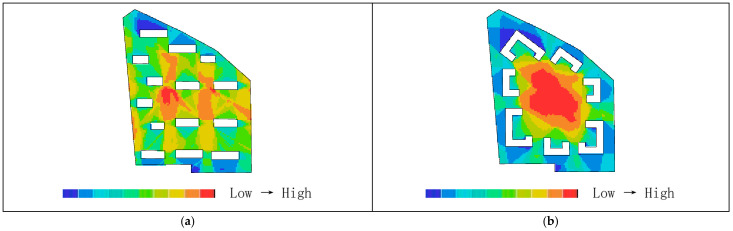
(**a**) Visual Integration of the Residential Area Before Transformation; (**b**) Visual Integration of the Residential Area After Transformation.

Areas with higher visual integration values are represented by warmer colors, while areas with lower values are shown in cooler colors. The visibility integration maps before and after transformation are shown in Figure 21a,b. As shown in Table 5, before transformation, the global integration ranges from 6.46 to 17.98, with an average value of 12.93 and a standard deviation of 2.46, indicating a relatively dispersed distribution of integration. After transformation, the global integration ranges from 6.54 to 24.44, with an average value of 15.21 and a standard deviation of 5.29. In both cases, the areas with the highest integration (warmest colors) are concentrated in the central region, while the areas with the lowest integration (coolest colors) are distributed toward the outer edges near residential buildings. A comparison of the mean and standard deviation of visual integration indicates that the transformed scheme exhibits higher overall integration, with a more concentrated and clearly hierarchical distribution. The central area demonstrates greater public openness and visual permeability. Visual integration presents a gradient distribution that decreases from the inside outward, which is highly consistent with the hierarchical structure and functional coordination of the nucleus–cytoplasm system, reflecting the spatial visibility and permeability of the biomimetic cellular structure.

**Figure 22:**
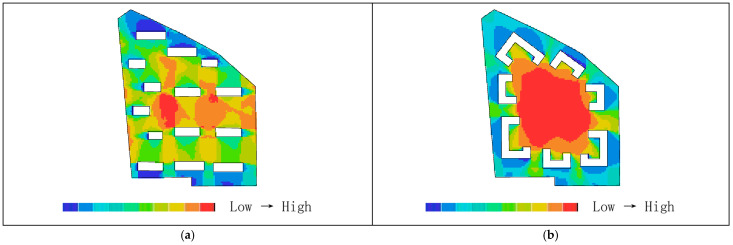
(**a**) Visual Connectivity of the Residential Area Before Transformation; (**b**) Visual Connectivity of the Residential Area After Transformation.

As shown in Table 4, before transformation, the visual connectivity ranges from 259 to 2866, with an average value of 1751.69. The warm-colored areas, representing higher visual connectivity values, are distributed along the central axis from the residential entrance to the central plaza. After transformation, the visual connectivity ranges from 103 to 3774, with an average value of 2233.33. The warm-colored areas, indicating higher visual connectivity, are predominantly distributed within the ring-shaped spaces enclosed by buildings. By comparing the average values of visual connectivity, it can be concluded that the residential area after transformation has higher connectivity and better spatial permeability. As shown in Figure 22a,b, areas with higher visual connectivity are represented by warmer colors, while areas with lower values are shown in cooler colors.

**Figure 23:**
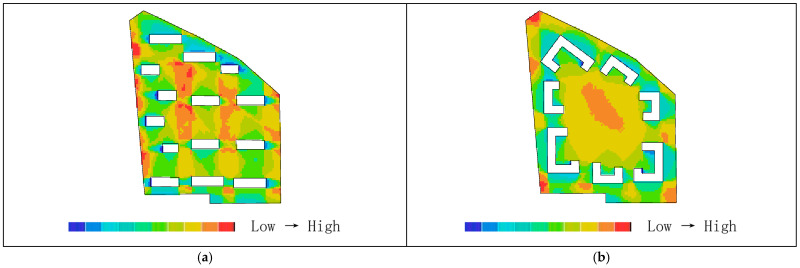
(**a**) Visual Control of the Residential Area Before Transformation; (**b**) Visual Control of the Residential Area After Transformation.

As shown in Figure 23a,b, the comparison of visual control values in the residential area indicates that regions with higher values are represented by warmer colors, while lower values correspond to cooler colors. As shown in Table 5, before transformation, the visual control values range from 0.22 to 1.46, with an average value of 1.00, primarily concentrated between buildings in the central area, with some higher values appearing at the eastern and western boundary corners. After transformation, the visual control values range from 0.07 to 1.56, with an average value of 1.00, mainly concentrated in the central area enclosed by buildings. This indicates that the overall control values before and after transformation are similar, while the post-transformation distribution is more extensive, and the central visual field is more optimal and more reasonable.

**Figure 24:**
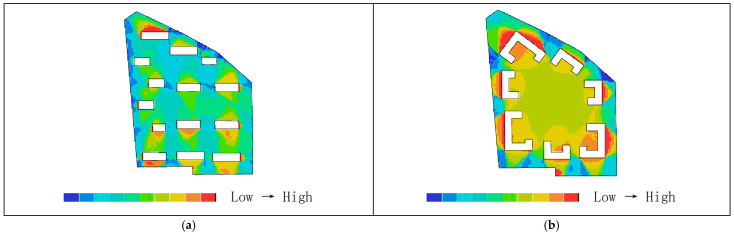
(**a**) Visual Clustering Coefficient of the Residential Area Before Transformation; (**b**) Visual Clustering Coefficient of the Residential Area After Transformation.

In the visual clustering coefficient map of the residential area, warm colors indicate higher clustering values and greater visual obstruction, while cool colors indicate lower clustering values and more open visibility. Figure 24a,b present a comparison of the visual clustering coefficients before and after transformation. As shown in Table 4, before transformation, the visual clustering coefficient ranges from 0.36 to 1.00, with an average value of 0.64. The warm-colored areas radiate outward from the center and are distributed within the shadow zones of each building, with the highest values observed particularly in the residential buildings on the northwest and southwest sides, indicating severe visual obstruction and stronger privacy. The cool-colored areas, representing more open visibility, are scattered along the boundary of the site. After transformation, the visual clustering coefficient ranges from 0.39 to 1.00, with an average value of 0.76. The warm-colored areas with the highest clustering values are mainly distributed in the regions enclosed by buildings. Therefore, the visual clustering coefficients before and after transformation are relatively similar; however, in both cases, the peripheral areas around buildings exhibit relatively limited visibility, with façade views being obstructed and constrained, resulting in insufficient openness but stronger privacy.

**Figure 25:**
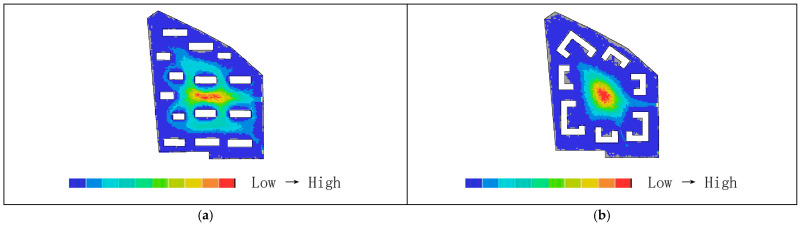
(**a**) Agent-Based Visibility Analysis of the Residential Area Before Transformation; (**b**) Agent-Based Visibility Analysis of the Residential Area After Transformation.

The experiment establishes two entrances on the eastern side of the residential area based on biomimetic planning design and conducts pedestrian flow simulation to quantitatively evaluate the frequency of spatial node usage and residents’ dwelling tendencies, thereby objectively revealing how spatial layout influences spontaneous activity patterns. As shown in Table 5, before transformation, the pedestrian flow ranges from −1 to 482, with an average value of 65.82 and a standard deviation of 84.67. Figure 25a shows that, before transformation, the warm-colored areas, indicating the highest pedestrian density, are concentrated in the central plaza, while the cool-colored areas, indicating sparse pedestrian flow, are distributed along the periphery of the residential area. Figure 25b shows that, after transformation, the pedestrian flow ranges from −1 to 639, with an average value of 62.75 and a standard deviation of 115.94. The warm-colored areas, representing the highest pedestrian density, are concentrated in the central public service facilities, while the cool-colored areas remain distributed along the periphery. This indicates that the average pedestrian flow values before and after transformation are similar, and the central areas in both cases exhibit good accessibility and permeability; however, the distribution difference is more pronounced after transformation, with a clearer hierarchical structure, and the pedestrian flow in the central public service facilities is higher than that in the pre-transformation plaza.

The visibility analysis indicates that, compared with the pre-transformation residential buildings, the orderly arrangement of buildings results in restricted visual fields, limiting the enjoyment of open landscapes and reducing the utilization and attractiveness of spaces located farther from the central area. After transformation, the residential buildings exhibit improved spatial permeability, facilitating the aggregation of people in the core area. The centripetal visual distribution toward public facilities becomes more balanced, well-organized, and hierarchically structured, although visibility remains limited on building façades facing away from public spaces. However, this is compensated for by landscape greenery in the peripheral areas.

## 4. Discussion

### 4.1. Integration of Biomimetic Design Methods and Space Syntax

This study takes cellular physiological structure and its functional coordination mechanisms as the prototype to construct a biomimetic design method for five-minute living circle residential areas. The core lies in achieving systematic optimization of urban spatial organization through the coupling logic of “structure–function–behavior.” Compared with traditional planning approaches dominated by functional zoning or traffic efficiency, this method no longer treats spatial elements as isolated units but considers the residential area as a “quasi-living system” with inherent organizational logic, thereby emphasizing the dynamic relationship among spatial structure, functional layout, and human behavior. From the perspective of biomimetic mechanisms, the study does not remain at the level of morphological analogy but carries out functional abstraction and cross-scale translation of cellular structures. According to the definition of biomimetics proposed by Fayemi et al., which involves the abstraction, transfer, and application of biological model knowledge to solve engineering problems [53], this study maps the cell membrane, nucleus, cytoplasm, and cytoskeleton to the boundary system, public core, environmental matrix, and transportation network of residential areas, respectively. This mapping not only reconstructs spatial form but, more importantly, introduces an operational mechanism analogous to “central control–material transport–structural support” in cellular systems, thereby forming a spatial organization with inherent synergy.

At the level of method integration, this study further combines the above biomimetic structural model with space syntax analysis to construct a research framework of “biomimetic structure generation–syntactic quantitative analysis–spatial performance feedback.” The introduction of space syntax enables the transition of biomimetic design from qualitative conception to quantitative verification. Specifically, the calculation of indicators such as integration, connectivity, and mean depth can reveal the potential mechanisms through which spatial structure influences human behavior. The results show that the introduction of the biomimetic cellular structure significantly increases spatial integration and reduces mean depth, indicating enhanced overall accessibility of the spatial system. Meanwhile, increases in connectivity and synergy indicate that the spatial network establishes a closer relationship between local and global structures. These changes quantitatively verify the effectiveness of the biomimetic structure in optimizing spatial organization. More importantly, this study goes beyond traditional morphological biomimicry, which remains limited to the static structural level of cells, by introducing dynamic mechanisms such as material transport, signal transmission, distributed metabolism, and adaptive homeostasis maintenance. The significant improvement in key indicators is thus attributed to the operational logic of cellular organization, distinguishing this model from conventional centralized planning modes.

In addition, the implementation of biomimetic methods relies on computational tools to analyze and integrate complex spatial relationships. Kruiper et al. pointed out that systematic biomimetic design requires computer-based tools to embed biological knowledge into engineering methodologies [54]. In this study, spatial syntax tools such as Depthmap are used to conduct multidimensional analysis of residential space, achieving deep integration between biomimetic design and digital analytical techniques. This not only improves the controllability of the design process but also enhances the reproducibility and scientific rigor of the research results. From a broader perspective, biomimetics, as an interdisciplinary approach, is gradually becoming an important pathway for promoting sustainable design. Related studies suggest that biomimetic design can provide inspiration for technological innovation and support sustainable development goals at ecological and environmental levels [55]. The biomimetic cellular planning method proposed in this study, by optimizing spatial structure and functional configuration, not only improves accessibility and spatial vitality in residential areas but also enhances environmental adaptability and ecological performance to a certain extent. This indicates that the application of biomimetic principles at the urban scale holds multiple values, ranging from spatial optimization to sustainable development.

The performance improvements achieved in this study, including increased spatial integration, reduced mean depth, and optimized accessibility and connectivity, can partly be realized through conventional planning strategies such as accessibility-oriented planning, center-aggregation planning, and road-network densification. For example, measures such as adding pedestrian passages, increasing the density of branch road networks, and centrally arranging public service facilities can all improve spatial topological performance to a certain extent. However, the unique value of the biomimetic cellular planning model lies in the fact that its optimization effects do not merely result from local facility adjustments or road-network densification, but from the systematic organizational logic generated by the coordinated operation of the cell membrane, cytoplasm, nucleus, organelles, and cellular microfilaments. The characteristics it presents—such as central gradient radiation, whole-area homogeneous permeation, distributed unit autonomy, flexible boundary control, and hierarchical efficient transmission—cannot be fully achieved through traditional incremental planning. Conventional planning is usually driven by a single objective, such as maximizing accessibility, and is prone to problems such as central overload, peripheral weakening, and disconnection between local and global structures. In contrast, the biomimetic cellular organizational logic aims at the synergistic homeostasis of living systems, thereby achieving the simultaneous optimization of global integration and local balance, the parallel enhancement of spatial efficiency and community vitality, and the unification of structural optimization and mechanism adaptation.

### 4.2. Spatial Accessibility and Urban Vitality of Five-Minute Living Circles Under Biomimetic Mechanisms

In the daily activities of residential areas within the living circle, walking is the most fundamental and primary mode of travel [56]. The accessibility of the pedestrian system largely reflects the influence of spatial structure on human behavior, essentially depending on the degree of spatial barriers within the network: the lower the barriers, the higher the accessibility, and the more frequent the movement and interaction of individuals within the space [57]. From the perspective of space syntax, accessibility is not only reflected in shorter path distances but also in increased spatial integration and connectivity, which directly affect the frequency of human presence, dwelling behavior, and path selection.

The results show that the residential area optimized based on the biomimetic cellular structure performs significantly better than the traditional layout mode in key indicators such as integration, connectivity, and mean depth. This optimization of spatial structure essentially reduces the “topological resistance” of the spatial system, enabling residents to reach target nodes through fewer spatial transitions, thereby improving the walkability and accessibility of the overall space. The high accessibility generated by spatial structural optimization allows public activity spaces to no longer rely on a single axis or entrance to organize pedestrian flow. Instead, balanced permeation is achieved through a multi-path network, whole-area resource distribution is realized through matrix-like permeation, and system homeostasis is maintained through feedback regulation. These dynamic processes are translated into a pedestrian-priority network, continuous landscape permeation, balanced radiation of public services, and adaptive boundary control in residential areas, thereby enhancing the overall operational efficiency of the spatial system. In the planning context of the five-minute living circle, the improvement of spatial accessibility is not merely a matter of traffic efficiency but also a fundamental mechanism for generating the vitality of public spaces. With a walking radius of approximately 300 m, the living circle organizes residents’ daily activities through the radiating effect of internal public spaces. Previous studies have shown that the frequency of human presence, movement, and dwelling at spatial nodes is an important indicator of accessibility [52]. When a space has higher integration and choice values, it is more likely to attract pedestrian aggregation, thereby forming public spaces with sustained vitality. This mechanism differs significantly from traditional planning approaches oriented toward functional zoning and traffic efficiency, which often overlook the role of spatial structure in guiding social behavior [58].

Urban vitality, as an important indicator of residential quality, is closely related to the intensity of activities in public spaces, particularly at the neighborhood and residential scales [59]. Traditional high-rise residential areas, due to their monotonous spatial structures and fragmented public spaces, often struggle to form stable social places, resulting in insufficient interaction among residents and weak community cohesion. In contrast, this study constructs a “core–ring–radial” spatial system through biomimetic cellular structures, placing public service facilities as the “cell nucleus” at the spatial center and connecting them through highly integrated road networks, enabling pedestrian flow to naturally converge in the core area, thereby significantly enhancing the intensity of public space use and social activity. Furthermore, spatial optimization also positively impacts the ecological and environmental quality of residential areas. Through the rational allocation of green spaces and landscape systems within the inner and outer rings, ecological service functions can be provided while spatial comfort and recognizability are also enhanced. In terms of microclimate regulation, for example, the biomimetic cellular model arranges gradient green belts and street-tree canopies in the inner and outer rings, which can reduce summer surface temperatures and extend the duration of residents’ outdoor activities. If the core activity area lacks shading or ventilation corridors, residents may avoid using the space during high-temperature periods even when its integration value is high, resulting in actual urban vitality being lower than the topological prediction. In terms of noise buffering, the ecologically friendly boundary in the cell membrane analogy, through C-shaped semi-enclosed building forms and multi-layered greenery, can reduce traffic noise along roadside areas, meet the standards of quiet zones, and significantly increase residents’ willingness to stay and the frequency of neighborhood interaction. In terms of landscape materials, the dynamic order and corner filling in the analogy between landscape environmental facilities and the cytoplasm are implemented through specific choices of paving texture and color: non-slip permeable wooden platforms or permeable bricks can create an inviting atmosphere and promote spontaneous social activities. Relevant studies indicate that the spatial vitality of urban green spaces has become an important dimension in evaluating public space quality [60]. The application of biomimetic cellular structures in five-minute living circles not only optimizes the physical spatial structure through improved space syntax indicators but also promotes the aggregation and interaction of human behavior by enhancing accessibility and spatial integration, thereby improving urban vitality and overall residential quality at a mechanistic level. This demonstrates that spatial organization models based on biomimetic principles can establish an effective coupling relationship among “spatial structure–behavior patterns–social vitality,” providing a theoretically robust pathway for optimizing high-density residential areas.

### 4.3. Transformation from Single-Cell to Multicellular Spatial Organization Structures and Application Prospects in Residential Area Planning

Using a single eukaryotic cell as the prototype to construct the spatial model of a five-minute living circle verifies the effectiveness of the biomimetic structure for an individual residential area. However, urban space is not an isolated cellular unit but a complex system composed of a large number of interlocking residential areas. Based on the biomimetic mechanisms of multicellular biological tissues, a preliminary theoretical framework can be inferred as follows. First, each living circle retains its own “nucleus”—namely, the public service facilities of the residential area—while a shared “regional nucleus,” such as a subdistrict service center, is established at the multicellular tissue level and connected through radial arterial roads. Second, the boundary zones between living circles are transformed from passive isolation belts into active interaction zones, where cross-circle shared community commerce or pocket parks are arranged as functional nodes of the multicellular organization. Third, space syntax indicators such as synergy (R^2^) and intelligibility are used for evaluation. When the global–local synergy of multiple living circles exceeds 0.7, it indicates that the spatial structure has achieved “tissue-level” integrity; otherwise, cross-living-circle road connections or functional coupling should be strengthened. Fourth, drawing on the plasticity of multicellular tissues, the boundaries of living circles are allowed to undergo dynamic adjustment in response to changes in population density, fluctuations in traffic flow, or urban renewal cycles. For example, convertible “boundary plots” may be reserved as green buffer zones in the initial stage and later developed into cross-living-circle service facilities. In this way, the expansion from a single five-minute living circle to the coordinated organization of multiple living circles can be achieved, providing a direction for subsequent empirical research on the interaction mechanisms among multiple adjacent living circles.

Based on the planning application prospects of transforming single-cell biomimetic logic into multicellular spatial organization structures, coordinated coupling across spatial scales can be achieved. The core value of the biomimetic cellular structure lies not only in the steady-state structure itself but, more importantly in dynamic life processes such as material transport, energy metabolism, information transmission, distributed regulation, and self-organized homeostasis maintenance. At the level of multicellular linkage, the five-minute living circle can further establish a life-like dynamic evolutionary mechanism: the hierarchical regulatory logic of the nucleus can be used to construct a coordinated transmission pathway between central services and district-level coordination; the matrix-permeation logic of the cytoplasm can realize the continuous provision of ecological systems, landscape structures, and public spaces across living circles; the distributed autonomy logic of organelle clusters can ensure the relative independence and efficient operation of residential area units; the network transport logic of cellular microfilaments can construct a hierarchical, resilient, and highly adaptable transportation and pedestrian system; and the selective permeability logic of the cell membrane can enable safe, orderly, and flexible boundary interaction among living circles. Through these mechanisms, multicellular spatial organization structures can become life-like systems with adaptive regulation, symbiotic coordination, organic renewal, and dynamic feedback capabilities. Consequently, biomimetic cellular planning can move beyond structural correspondence and functional matching, truly advancing toward the higher-level biomimetic principles of process biomimicry, system biomimicry, and evolutionary biomimicry, thereby significantly enhancing the theoretical depth of the research.

### 4.4. Limitations and Future Research Directions of the Biomimetic Planning Method

Although this study constructs a spatial organization model for five-minute living circle residential areas based on biomimetic principles derived from cellular structures and quantitatively verifies its spatial performance through space syntax methods, several limitations remain that require further investigation. First, at the level of biomimetic translation, this study constructs a spatial model based on the analogical relationship between cellular structure and function. Although cross-scale mapping from biological systems to urban space has been achieved, the translation of dynamic regulatory mechanisms in biological systems, such as feedback regulation [61] and self-organized evolution, still requires further elaboration. High-level biomimetic design should shift from static morphological imitation toward dynamic mechanism simulation in order to enhance the adaptability and evolutionary capacity of the system [62]. Second, in terms of methodological application, space syntax, as a spatial analysis tool based on topological relationships, can effectively reveal the potential influence of spatial structure on human behavior. However, its interpretive framework still relies mainly on spatial morphological variables and has limited capacity to analyze non-topological relationships such as microclimate, noise, and landscape materials. Relying solely on spatial structural indicators is insufficient to fully explain the formation mechanism of urban spatial vitality [63]. Therefore, comprehensive analysis should be conducted by integrating multi-source data, such as mobility trajectories and big-data sensing, with environmental simulation methods [64]. In addition, this study conducts empirical analysis based on a single case and does not empirically examine dynamic coordination, metabolic interaction, or self-organized evolution among multiple living circles. Although the effectiveness of the biomimetic cellular structure in a specific context can be verified, its applicability under different urban forms, density conditions, and social backgrounds still requires further examination. The complexity of urban systems determines that spatial optimization strategies are highly context-dependent, and comparative studies across multiple cases are considered a key approach to enhancing the external validity of planning methods [65].

The biomimetic cellular structure design principles proposed in this study are mainly grounded in the qualitative translation from biological mechanisms to spatial organization rules, as well as morphological–functional analogy. The introduction of complex-system concepts, such as self-organization, distributed coordination, and dynamic regulation, represents a functional abstraction of and design inspiration from the operating principles of eukaryotic cells. The focus lies in constructing a qualitative translation framework linking cellular structures, spatial rules, planning interventions, and syntactic performance, and in conducting quantitative verification through space syntax. Spatial performance is objectively evaluated using topological indicators such as integration, connectivity, mean depth, and synergy, thereby confirming the optimization effects of the biomimetic cellular model compared with traditional layouts. At present, these dynamic mechanisms have not yet been independently quantitatively verified or dynamically simulated through methods such as multi-agent behavioral simulation, evolutionary computation, or dynamic pedestrian flow simulation. The dynamic response characteristics, adaptive regulation capacity, and long-term evolutionary patterns of the relevant mechanisms remain to be further examined in future research through quantitative simulation and empirical observation. Therefore, the discussion of these mechanisms in this paper is primarily based on qualitative interpretation and theoretical inference, while the research conclusions focus on the optimization effects of spatial topological structure. The quantitative verification of the related theoretical assumptions will be further deepened as a core direction of future research.

From the perspective of practical implementation and technological development, the application of biomimetic cellular planning in existing urban environments still faces real-world constraints, including land use conditions, development intensity controls, and path dependence of existing transportation systems. Its feasibility in urban regeneration contexts requires further exploration [66]. With the advancement of computational design and artificial intelligence technologies, biomimetic urban planning is gradually shifting from experience-driven approaches toward data-driven and generative mechanism-oriented paradigms. Related studies have attempted to simulate the generation of complex spatial systems through parametric modeling and multi-objective optimization methods, thereby improving the adaptability and performance of design solutions [67]. Future research should embed the organizational logic of biomimetic cellular structures into computational generative frameworks, enabling the transition from conceptual design to computable and optimizable design methodologies. Combined with multi-source data and multi-scale case validation, this approach can further enhance the scientific explanatory power and practical applicability of biomimetic planning methods. Biomimetic principles still hold broad potential for application in urban spatial design, and their deep integration with quantitative analytical methods and digital technologies will become an important direction for optimizing high-density residential environments.

## 5. Conclusions

This study takes the organizational mechanism of biological cell structures as a prototype to construct a biomimetic cellular spatial organization model for five-minute living circle residential areas and quantitatively evaluates its spatial performance using space syntax methods. Based on comparative analysis of the case study, the results indicate that the biomimetic cellular structure demonstrates systematic optimization across key syntactic indicators: global integration increases from 1.27 to 1.64 (an increase of approximately 29.1%), mean depth decreases from 3.79 to 3.18 (a reduction of approximately 16.1%), spatial connectivity increases from 3.74 to 5.44 (an increase of approximately 45.5%), and axial synergy increases from 0.44 to 0.86, indicating significant improvements in spatial centrality, accessibility, and the relationship between global and local structures at the topological level. Meanwhile, the spatial organization shifts from a single linear structure to a multi-level network system characterized by “core–ring–radial” patterns, resulting in a gradient distribution of spatial integration from the core to the periphery. This effectively reconstructs the distribution of pedestrian flow and enhances the agglomeration effect of central public spaces. These quantitative results validate, from a mathematical perspective, the effectiveness of the biomimetic cellular structure in improving spatial structural efficiency and enhancing spatial vitality. By establishing a research framework of “biomimetic structure generation–syntactic quantification–spatial performance feedback,” this study achieves cross-scale mapping from biological structures to urban space and provides a quantifiable and verifiable biomimetic design approach for optimizing high-density residential areas.

Although this study verifies the effectiveness of the biomimetic cellular planning model in optimizing a single five-minute living circle, certain limitations remain. The research mainly focuses on a single living circle and does not conduct empirical analysis of the interaction mechanisms among multiple adjacent living circles. Meanwhile, the space syntax method emphasizes the analysis of spatial topological relationships and has limited capacity to examine non-topological factors such as microclimate, noise, and landscape materials. Future research will further validate the findings through cross-circle space syntax analysis, multi-agent simulation, and field investigation. Multi-source spatiotemporal behavioral data and environmental simulation methods, such as microclimate simulation and acoustic analysis, will be introduced to construct a comprehensive analytical framework coupling spatial structure and human behavior. Furthermore, parametric design and generative algorithms will be integrated to continuously promote the transformation of biomimetic cellular structures from static models toward dynamic generative mechanisms. Ultimately, by expanding empirical research across different urban contexts, the application of biomimetics in the design of urban human settlements can be extended, thereby improving its applicability and potential for broader implementation in complex urban renewal and residential area planning.

## Figures and Tables

**Table 1:** Biomimetic Translation of Cellular Structures and Space Syntax Outcomes.

Cellular Structure	Core Biological Mechanism	Translated Spatial Rules	Corresponding Planning Intervention Measures	Expected Space Syntax Outcomes
Cell membrane	Selective permeability, physical barrier, signal recognition, dynamic regulation	Flexible boundary control; orderly interaction between internal and external spaces	Ecological hedge boundaries, intelligent access-controlled entrances and exits, noise buffer zones, visually and ventilatively permeable interfaces	Improved connectivity of boundary spaces; reduced mean depth in peripheral areas; enhanced system intelligibility and safety
Cytoplasm	Whole-area homogeneous permeation, matrix support, microenvironmental regulation, dynamic buffering	Continuous landscape coverage; corner-space filling; homogeneous distribution of public spaces	Whole-area green permeation, landscaping of idle plots, buffer green spaces between clusters, microclimate-adaptive landscape layout	Overall improvement in spatial connectivity; balanced distribution of local integration; significant improvement in visual integration and spatial permeability
Nucleus	Central regulation, signal radiation, resource coordination, genetic information control	Centralized core services; 300 m radius-based radiation; coordinated functional scheduling	Layout of public service centers, radial service paths, core open spaces, aggregation of one-stop service facilities	Significant improvement in global integration; increased choice and control values in the core area; enhanced spatial aggregation effect
Organelle clusters	Functional specialization, distributed autonomy, unit coordination, locally efficient operation	Functional differentiation of residential clusters; semi-enclosed layout; centripetal organization	C-shaped semi-enclosed residential buildings, 300 m centripetal clusters, orthogonal road connections, distributed convenience service nodes	Balanced distribution of local integration; improved connectivity among clusters; enhanced system synergy and local operational efficiency
Cellular microfilaments	Structural support, directional material transport, hierarchical network, dynamic circulation guidance	Pedestrian priority; hierarchical road network; ring-shaped and radial layout; internal–external connectivity	Three-level road system, ring-shaped main roads, radial pedestrian paths, separation of pedestrian and vehicular traffic, rapid emergency access routes	Optimized road integration and choice; reduced mean depth; substantial improvement in axial synergy and spatial intelligibility

**Table 3:** Quantitative Results of Convex Space Analysis.

**Convex Space Analysis Before Transformation**	**Maximum**	**Minimum**	**Mean**	**Standard Deviation**
Global Integration	1.52	0.88	1.27	0.17
Local Integration	2.47	1.00	1.74	0.32
Mean Depth	4.17	3.23	3.791	0.39
Connectivity	9.00	1.00	3.74	1.87
**Convex Space Analysis After Transformation**	**Maximum**	**Minimum**	**Mean**	**Standard Deviation**
Global Integration	2.25	1.16	1.64	0.22
Local Integration	2.75	1.54	2.12	0.27
Mean Depth	4.03	2.57	3.18	0.29
Connectivity	14.00	2.00	5.44	2.20

**Table 4:** Quantitative Results of Axial Analysis.

**Axial Analysis Before Transformation**	**Maximum**	**Minimum**	**Mean**	**Standard Deviation**
Global Integration	0.84	0.48	0.65	0.08
Local Integration	2.25	0.58	1.13	0.30
Choice	750.00	0	203.41	196.58
Control Value	5.09	0.14	1.00	0.86
**Axial Analysis After Transformation**	**Maximum**	**Minimum**	**Mean**	**Standard Deviation**
Global Integration	2.10	0.88	1.33	0.28
Local Integration	2.40	1.04	1.57	0.30
Choice	480.00	0	88.10	126.84
Control Value	3.77	0.13	1.00	0.93

**Table 5:** Quantitative Results of Visibility Analysis.

**Visibility Analysis Before Transformation**	**Maximum**	**Minimum**	**Mean**	**Standard Deviation**
Global Integration	17.98	6.46	12.93	2.46
Connectivity	2866	259	1751.69	640.26
Control Value	1.46	0.22	1.00	0.21
Clustering Coefficient	1.00	0.36	0.64	0.11
Agent	482.00	−1	65.83	84.67
**Visibility Analysis After Transformation**	**Maximum**	**Minimum**	**Mean**	**Standard Deviation**
Global Integration	24.44	6.54	15.21	5.29
Connectivity	3774	103	2233.33	1185.55
Control Value	1.56	0.07	1.00	0.24
Clustering Coefficient	1.00	0.394	0.76	0.13
Agent	639	−1	62.08	115.94

## Data Availability

The original contributions presented in this study are included in the article. Further inquiries can be directed to the corresponding author.
